# Study on Loading of Na_2_WO_4_ and Silanization Treatment on Surface of Plasma Electrolytic Oxidation Coatings with Different Structures

**DOI:** 10.3390/ma18174146

**Published:** 2025-09-04

**Authors:** Donghao Lei, Ziyi Wang, Jinjun Qiao, Lingyun An, Chenggong Chang, Leichao Meng, Zhanying Wang, Yanping Yang

**Affiliations:** 1 Qinghai Provincial Key Laboratory of Nanomaterials and Technology, School of Chemistry and Materials Science, Qinghai Minzu University, Xining 810007, China; 18822203344@163.com (D.L.); zy2534929264@126.com (Z.W.); 13709765574@163.com (J.Q.); leichao5166@163.com (L.M.); 15193195979@163.com (Z.W.); 15111799765@163.com (Y.Y.); 2 Key Laboratory of Comprehensive and Highly Efficient Utilization of Salt Lake Resources, Qinghai Institute of Salt Lake, Chinese Academy of Sciences, Xining 810008, China; 3 Key Laboratory of Salt Lake Resources Chemistry of Qinghai Province, Xining 810008, China

**Keywords:** magnesium alloy, plasma electrolytic oxidation, microstructure, voltage, Na_2_WO_4_, silanization treatment, corrosion resistance

## Abstract

To explore the influence of the microstructure of plasma electrolytic oxidation (PEO) coating on the loading of corrosion inhibitors and the silanization treatment on its surface, PEO coatings were first prepared on the surface of AZ31B magnesium alloy under different voltages. Secondly, sodium tungstate (Na_2_WO_4_) was loaded into the micropores and onto the surface of the PEO coatings via vacuum impregnation, and which were subsequently subjected to silanization treatment. The phase composition of the coatings was studied by XRD, while the elemental composition and valence state were investigated by XPS. The surface and cross-sectional morphology of the coatings, as well as the composition and distribution of elements, were studied by SEM and EDS. Image J software was employed to analyze the thickness of the coatings. The results show that the microstructure of PEO coatings prepared under different voltages varies, which affects the loading of Na_2_WO_4_ on the surface of PEO coating and the sealing effect of silanization treatment, thereby influencing the corrosion resistance of the coatings. As the voltage increases, the coating thickness and roughness gradually increase, while the surface porosity first increases and then decreases, and the loaded content of Na_2_WO_4_ also follows a trend of first increasing and then decreasing. Meanwhile, at 300 V and 350 V, silanization treatment effectively seals the PEO coatings loaded with Na_2_WO_4_. However, when the voltage increases to 400 V, due to the uneven surface of the PEO coating, nonuniform distribution of micropores, and high roughness, the silanization treatment fails to completely cover the coating. This results in defects such as pits on the surface of the composite coating prepared at 400 V. Therefore, the composite coating prepared at 350 V exhibits the best corrosion resistance. After immersion in a 3.5 wt.% NaCl solution for 240 h, the composite coating formed at 350 V remains intact, and its low-frequency impedance modulus |Z|_0.01Hz_ is as high as 1.06 × 10^6^ cm^2^. This value is approximately two orders of magnitude higher than that of the composite coating fabricated at 400 V and about three orders of magnitude higher than that of the pure PEO coating prepared at 350 V.

## 1. Introduction

Magnesium and its alloys occupy a significant position in the high-end manufacturing field owing to their advantages such as light weight and high performance. However, the issue of corrosion resistance of magnesium and its alloys still needs to be gradually addressed through material innovation, process optimization, and surface modification technologies [1,2,3]. Plasma electrolytic oxidation (PEO), also known as micro-arc oxidation (MAO), is a novel surface-modification technology that can grow a ceramic-like coating in situ on the metal surface, notable for the simple process, environmentally friendly electrolyte, excellent performance of the formed coating, and strong bonding force with the substrate. It is widely used in aerospace, the automotive industry, medical devices, electronics, and new energy fields [4,5,6]. However, the prepared coating inevitably has micropores and microcracks, which limit the improvement effect on the corrosion resistance of magnesium and its alloys.

To address the micropores and other defects in the PEO coating, scholars usually adopt post-treatment techniques for sealing [7,8]. The post-treatment techniques can be classified into three major categories: chemical sealing, physical sealing, and composite sealing [9,10]. Among them, silanization treatment, as one type of chemical sealing, is favored by researchers due to its advantages such as the simple process, good uniformity, excellent sealing effect, and strong bonding force with the coating. For example, Junchi Liu et al. [11] separately used KH560 (*γ*-Glycidoxypropyltrimethoxysilane), BTSE (1,2-bis(trimethoxysilyl)ethane), and DTMS (dodecyltrimethoxysilane) silane coupling agents as precursors to prepare silane films on the surface of AZ91D magnesium-alloy PEO coatings. The results showed that the silane films could effectively seal the defects in the PEO coating and significantly improved its corrosion resistance. In particular, KH560 and DTMS silanization treatments exhibited a more notable enhancement in the corrosion resistance of the PEO coating. Telmenbayar L et al. [12] prepared a 3-glycidyloxypropyltrimethoxysilane (GPTMS) layer on lanthanum-doped PEO coatings and found that the GPTMS layer further improved the corrosion resistance of the PEO coating. Huang J F et al. [13] prepared a double-layer micro-arc oxidation/3-glycidoxypropyltrimethoxysilane (KH560) composite film on the surface of LA103Z Mg-Li alloy via micro-arc oxidation and silanization treatments, and used reduced graphene oxide (RGO) to modify the KH560 film. The results showed that the prepared silane film was uniform and complete, filling the micropores and microcracks of the PEO coating and enhancing the shielding effect of the film. Moreover, RGO modification increased the thickness of the KH560 film, and the corrosion resistance of the composite coating was significantly improved.

In recent years, some researchers have also taken advantage of defects such as micropores and microcracks in PEO coatings to serve as loading containers for corrosion inhibitors [14,15,16]. For example, Yue Gong et al. [17] immersed a porous PEO coating in a Ce(NO_3_)_3_ solution to fabricate a novel CeO_2_-sealed PEO coating. It was observed that the incorporation of CeO_2_ effectively filled the micropores in the PEO coating, reducing its porosity and enhancing corrosion resistance. Moreover, the micropores in the PEO coating act as micro-containers for loading corrosion inhibitors, endowing the coating with self-healing properties and further improving its corrosion resistance. Mesoporous silica nanocontainers (MSNs) encapsulated with sodium benzoate (SB) corrosion inhibitors were strategically incorporated into the micropores of PEO coatings and the top epoxy (EP) layer by Ai-meng Zhang et al. [18]. The experimental results indicated that the distribution of these nanocontainers within the PEO micropores shortened the distance between MSN@SB and the substrate. This spatial proximity contributed to a low admittance value, thereby endowing the coating system with significant corrosion inhibition performance and excellent self-healing capability. Sajjad Akbarzadeh et al. [19] prepared smart coating systems on the PEO layer in which 8-hydroxyquinoline (8-HQ) and benzotriazole (BTA) were employed as corrosion inhibitive layers, followed by a sol–gel sealing. The results show that the PEO porosity can be considered natural ‘cages’ for surface impregnation/sealing, and the higher the concentration of the inhibitive layer, the lower the porosity.

In our previous paper, it was found that filling Na_2_WO_4_ into the micropores and onto the surface of the PEO coating can effectively improve the corrosion resistance of the PEO coating [20]. However, voltage, as one of the main parameters in the PEO coating preparation process, directly affects the porosity, thickness, and compactness of the PEO coating [21,22]. Consequently, the microstructure of PEO coatings prepared under different voltage conditions will vary, which will in turn affect the loading efficiency of subsequent corrosion inhibitors and the sealing effect of silanization.

Moreover, our previous research has also revealed that when different concentrations of Na_2_WO_4_ are vacuum-impregnated into the micropores and onto the surface of the plasma electrolytic oxidation (PEO) coating on magnesium alloy, followed by silanization treatment, the concentration of Na_2_WO_4_ impregnation significantly affects the sealing effect of the silanization treatment on the PEO coating—thereby influencing the overall corrosion resistance of the composite coating. When the Na_2_WO_4_ impregnation concentration is lower than 0.1 mol/L, the silane coating fails to effectively seal the surface pores of the PEO coating; when the impregnation concentration exceeds 0.1 mol/L, although effective sealing of the PEO coating pores can be achieved, the thickness of the formed silane coating is only at the nanoscale [20].

Therefore, this study aims to prepare PEO coatings with different microstructures by varying the PEO voltage. Subsequently, a 0.125 mol/L Na_2_WO_4_ corrosion inhibitor was vacuum-impregnated into the micropores and onto the surface of the PEO coatings. Meanwhile, the aging time of the silane solution was extended to 30 h. In accordance with the silanization procedure outlined in our previous work [20], a secondary silanization treatment was further conducted. The effects of the PEO coating microstructure on Na_2_WO_4_ loading content and silanization efficiency were investigated. Correlations among the PEO coating microstructure, corrosion inhibitor loading, and silanization sealing efficiency were established. This provides guidance for the preparation of PEO-based composite coatings with high corrosion resistance and excellent self-healing capability.

## 2. Experiment and Method

### 2.1. Materials and Reagents

In this experiment, AZ31B magnesium-alloy disks with a diameter of 45 mm and a thickness of 3 mm were selected as the substrates, supplied by Jiasheng Chemical Co., Ltd. (Lanzhou, China). Their chemical composition is 3.1 wt.% Al, 0.8 wt.% Zn, 0.15 wt.% Mn, 0.06 wt.% Si, 0.03 wt.% Cu, 0.02 wt.% Ni, 0.01 wt.% Fe, with the balance being Mg.

The reagents used in the experiment, namely Na_2_SiO_3_·9H_2_O (analytical grade), NaOH (≥96%), Na_2_WO_4_·2H_2_O (≥99.5%), NaCl (≥99.5%), and anhydrous ethanol (C_2_H_5_OH, ≥99.7%), were all purchased from Sinopharm Chemical Reagent Co., Ltd. (Shanghai, China). KF (99%) was obtained from Anhui Zesheng Technology Co., Ltd. (Anqing, China) and 3-glycidyloxypropyltrimethoxysilane (GPTMS, ≥97%) was sourced from Shanghai Aladdin Biochemical Technology Co., Ltd. (Shanghai, China).

### 2.2. Preparation of PEO Coatings with Different Microstructures

The magnesium-alloy substrates were successively polished with 400#, 800#, 1200# and 2000# silicon carbide sandpapers, ultrasonically cleaned with anhydrous ethanol for 10 min, and then dried with a cold air blower. The RZWH20A-II bipolar pulsed micro-arc oxidation power supply (Rizhao microarc technology Co., Ltd, Rizhao, China) was employed to treat the processed AZ31B magnesium-alloy substrate. The electrolyte composition consisted of Na_2_SiO_3_ at a concentration of 15 g/L, NaOH at 8 g/L, and KF at 8 g/L. It was prepared to a total volume of 4 L and stirred for 30 min to ensure the components were completely dissolved. Treatments were conducted at applied voltages of 300 V, 350 V, and 400 V, with a pulse frequency of 1000 Hz and a duty cycle of 12%. Each treatment process was endured for a duration of 10 min. During the preparation of the PEO coating, a magnetic stirrer was used to continuously stir the electrolyte to keep it in a flowing state, ensuring the uniformity of the electrolyte during the coating formation process. A cooling device was employed to maintain the electrolyte temperature at approximately 20 °C to prevent the electrolyte from deteriorating due to excessive heat. The PEO coatings prepared at voltages of 300 V, 350 V and 400 V were named 300-P, 350-P, and 400-P, respectively, as listed in Table 1.

### 2.3. Preparation of Na_2_WO_4_ Solution and Loading of Na_2_WO_4_

A 0.125 mol/L (41.231 g/L) aqueous solution of Na_2_WO_4_ was prepared using deionized water as the solvent. The mixture was stirred with a magnetic stirrer at room temperature for 30 min to ensure that the solute was completely dissolved. Its pH was adjusted to 11 using a 2 mol/L (80 g/L) NaOH solution. Then, PEO coatings with different microstructures were immersed in the prepared 0.125 mol/L Na_2_WO_4_ aqueous solution. The samples were vacuum-impregnated for 6 h in a vacuum-drying oven at 60 °C, followed by being blow-dried with a hair dryer, so that Na_2_WO_4_ could be loaded onto the micropores and surface of the PEO coatings.

### 2.4. Preparation of Silane Solution and nP-W-SG Composite Coatings

A silane solution was prepared by mixing anhydrous ethanol, 3-glycidyloxypropyltrimethoxysilane (GPTMS), and deionized water in a volume ratio of 45.3:33.7:21.0 (with a total volume of 100 mL), followed by stirring at room temperature for 1 h.

The PEO coatings loaded with Na_2_WO_4_ were immersed in the silane solution using a SYDC-100 dip coater (Three research technology Co., Ltd., Shanghai, China) at a speed of 100 mm/min for 15 min, then lifted at the same speed and allowed to cure at room temperature for 5 min. This operation was repeated three times. The coatings were then transferred to an oven, where they were cured at 60 °C for 1 h and subsequently at 100 °C for 24 h. After this process, they were taken out to cool down at room temperature, thus completing the initial silanization treatment of the Na_2_WO_4_-loaded PEO coatings.

**Figure 1:**
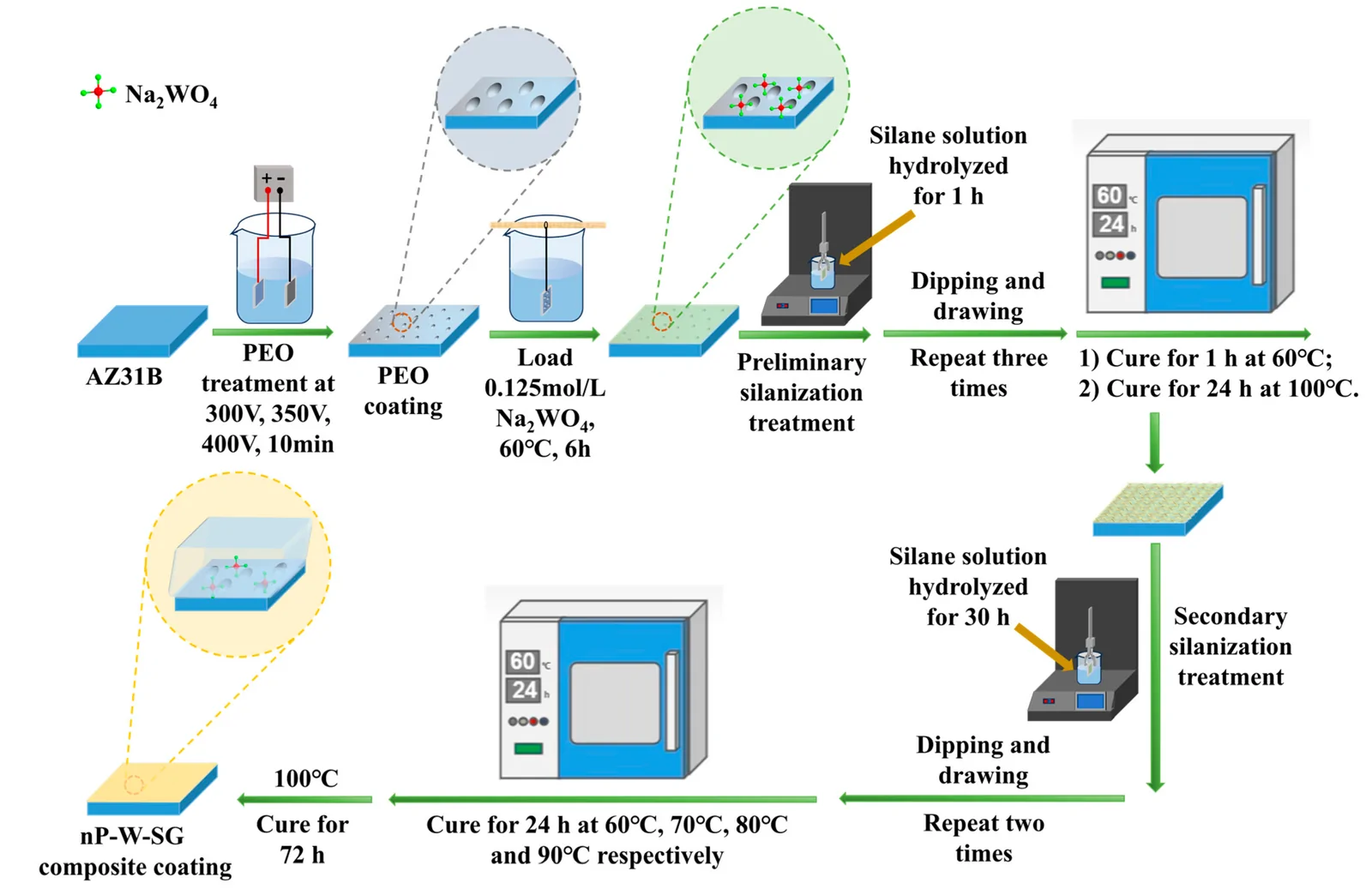
Process flow diagram of preparation of nP-W-SG composite coating.

After the silane solution was further aged at room temperature for 30 h, a secondary silanization treatment was applied to the Na_2_WO_4_-loaded PEO coatings that had undergone the initial silanization treatment, using a SYDC-100 dip coater (Three research technology Co., Ltd., Shanghai, China). The specific operation is as follows: The PEO coatings that had undergone the initial silanization treatment were immersed in a silane solution (which had been cured for 30 h) at a speed of 100 mm/min for 2 min. Then, the coatings were lifted at the same speed and left to stand for 1 min. This operation was repeated twice. Subsequently, they were transferred to a forced-air drying oven and cured at 60 °C, 70 °C, 80 °C, and 90 °C for 24 h, respectively, followed by curing at 100 °C for 72 h to ensure the complete drying and curing of the silane coating. Then, they were taken out and cooled down at room temperature to obtain the magnesium-alloy PEO composite coating (nP-W-SG). The process flow diagram for the preparation of the nP-W-SG composite coatings is shown in Figure 1, and the specific naming of the obtained composite coatings is listed in Table 1. Here, 300P-W-SG represents the composite coating obtained by loading Na_2_WO_4_ onto the surface of the 300-P coating (which was prepared at 300 V) followed by silanization treatment. Similarly, 350P-W-SG and 400P-W-SG denote the composite coatings prepared at 350 V and 400 V, respectively.

### 2.5. Characterization

The surface morphology of the coating was investigated by scanning electron microscopy (SEM, Thermo Fisher Scientific-Apreo 2C, Waltham, MA, USA and Japan JEOL-JSM-IT700HR, Tokyo, Japan). The chemical composition and elemental distribution on the surface and cross-section of the coating were analyzed by the energy-dispersive X-ray spectroscopy (EDS). The thickness of the coating was measured using Image J software (v1.52). The phase composition of the coating was detected by X-ray diffraction (XRD, Japan Rigaku Ultima IV, Tokyo, Japan). The chemical composition and valence state of the coating surface were studied by X-ray photoelectron spectroscopy (XPS, Thermo Scientific-ESCALAB Xi+, Waltham, MA, USA). The fine elemental spectra were fitted via peak-fitting using Avantage 5.948 software.

### 2.6. Performance Test

The corrosion resistance of PEO and its composite coatings was evaluated in a 3.5 wt.% NaCl solution using the CHI660E electrochemical workstation (Chenhua Instrument Co., Ltd., Shanghai, China) equipped with a standard three-electrode system. The working electrode was a PEO coating or composite coating with an exposed area of 1 cm^2^, and the reference electrode was a saturated calomel electrode, while the counter electrode was a platinum sheet. The initial potential was the open-circuit potential, the frequency range was 10^5^ to 10^−2^ Hz, the perturbation potential was 10 mV, and the tests were conducted successively for soaking times of 10, 48, 96, 144, 192, and 240 h. For each coating, at least three electrochemical tests were performed to ensure the reproducibility of the results. Data processing and the electrochemical impedance spectra (EIS) fitting were conducted via the complex nonlinear least-squares method with the aid of ZSimpWin 3.2 software. The fitting quality of impedance data for the selected equivalent circuits was evaluated based on two criteria: first, the χ^2^ values (which were required to be <0.01); and second, the frequency-dependent error distribution, determined by comparing experimental data with model-predicted data [23,24]. DRT analysis was carried out using MATLAB 2023 with DRTtools, with Gaussian functions selected as the basis functions for fitting calculations. The DRT data were peak-divided using Origin 2024 to obtain the area ratio of each peak.

## 3. Results

### 3.1. Phase Composition

**Figure 2:**
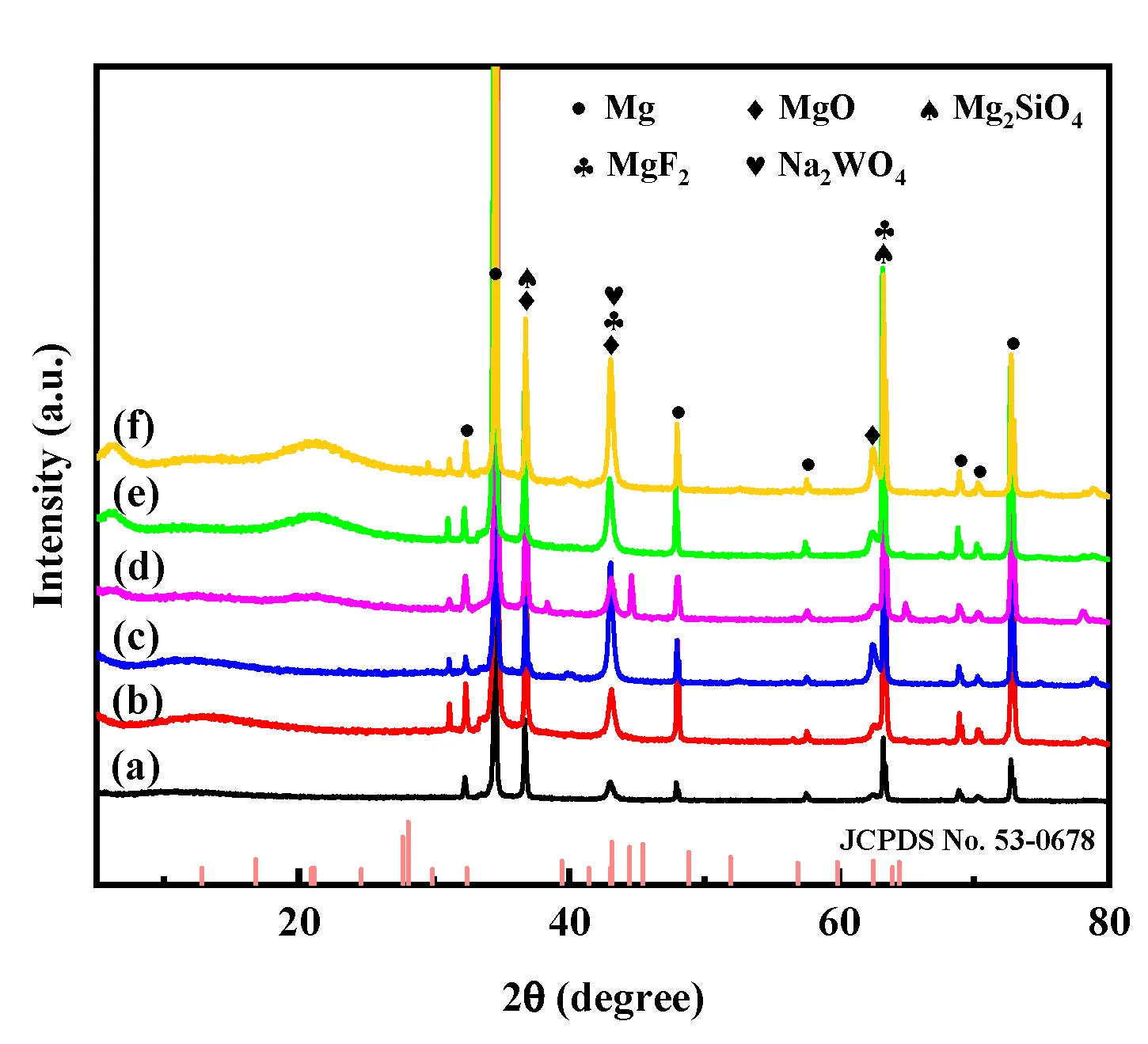
XRD patterns of different coatings: (**a**) 300-P; (**b**) 350-P; (**c**) 400-P; (**d**) 300P-W-SG; (**e**) 350P-W-SG; (**f**) 400P-W-SG.

Figure 2 shows the phase composition of PEO and its composite coatings prepared under different voltages. From Figure 2, it can be seen that the PEO coatings prepared under different voltages are mainly composed of phases such as Mg, MgO, Mg_2_SiO_4_, and MgF_2_, among which the diffraction peaks of Mg originate from the matrix. This indicates that the voltage does not change the phase composition of the PEO coatings. By comparing the 300-P, 350-P, and 400-P coatings, it can be found that with the increase in applied voltage, the intensity of the diffraction peak at 43.1° increases, indicating that the contents of phases such as MgO and MgF_2_ increase, which is consistent with the results reported by Liang [25] and Lee [26].

After loading Na_2_WO_4_ onto the micropores and surface of PEO coatings and performing silanization treatment, the obtained composite coatings contain the Na_2_WO_4_ phase (JCPDS No. 53-0678). When compared with the PEO coatings prepared under the same voltage conditions, the composite coatings obtained under different voltages exhibit a slight increase in the intensity of the diffraction peak at 43.1°. This phenomenon is attributed to the presence of Na_2_WO_4_ on the surface and within the micropores of the PEO coatings.

### 3.2. XPS Analysis

**Figure 3:**
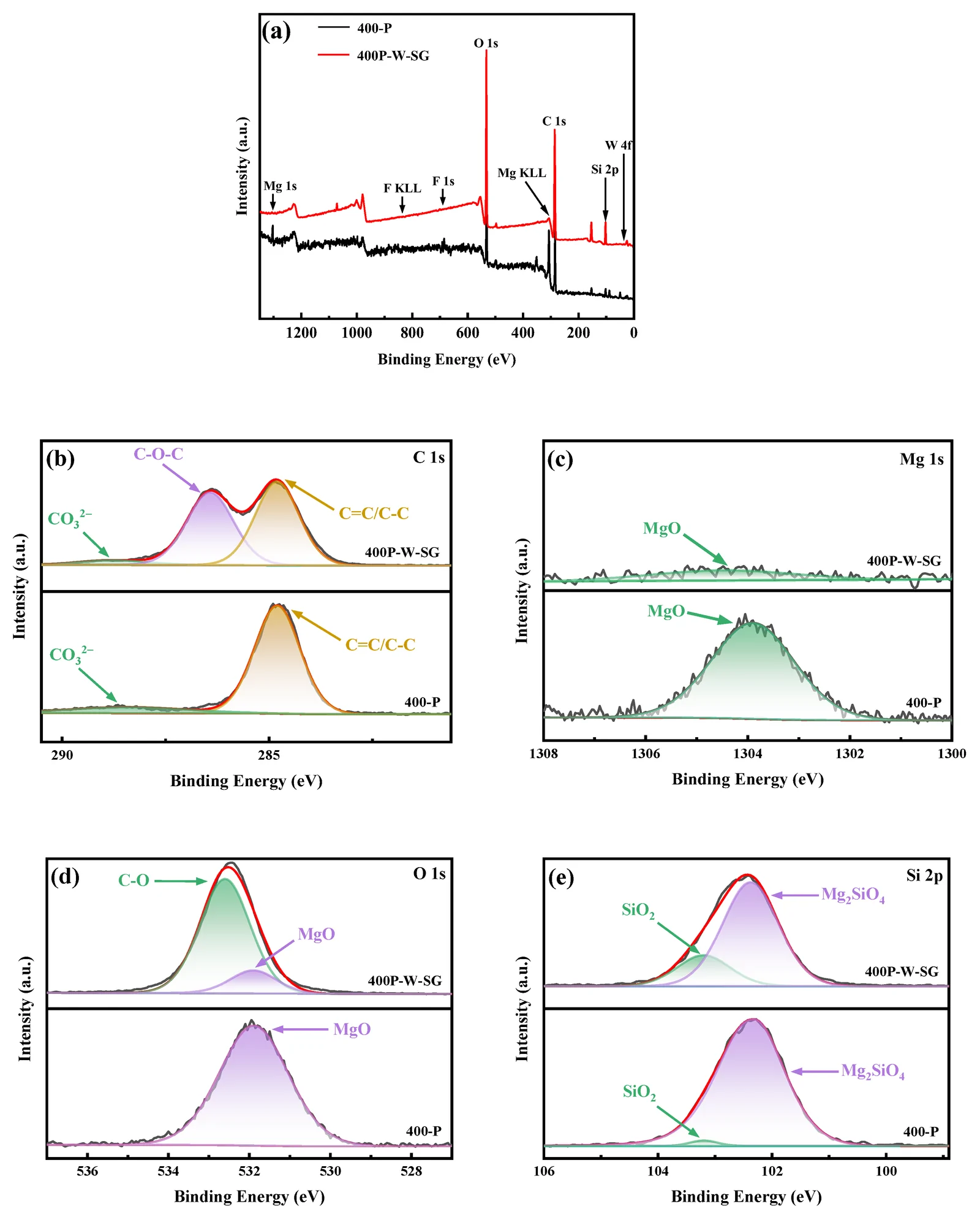

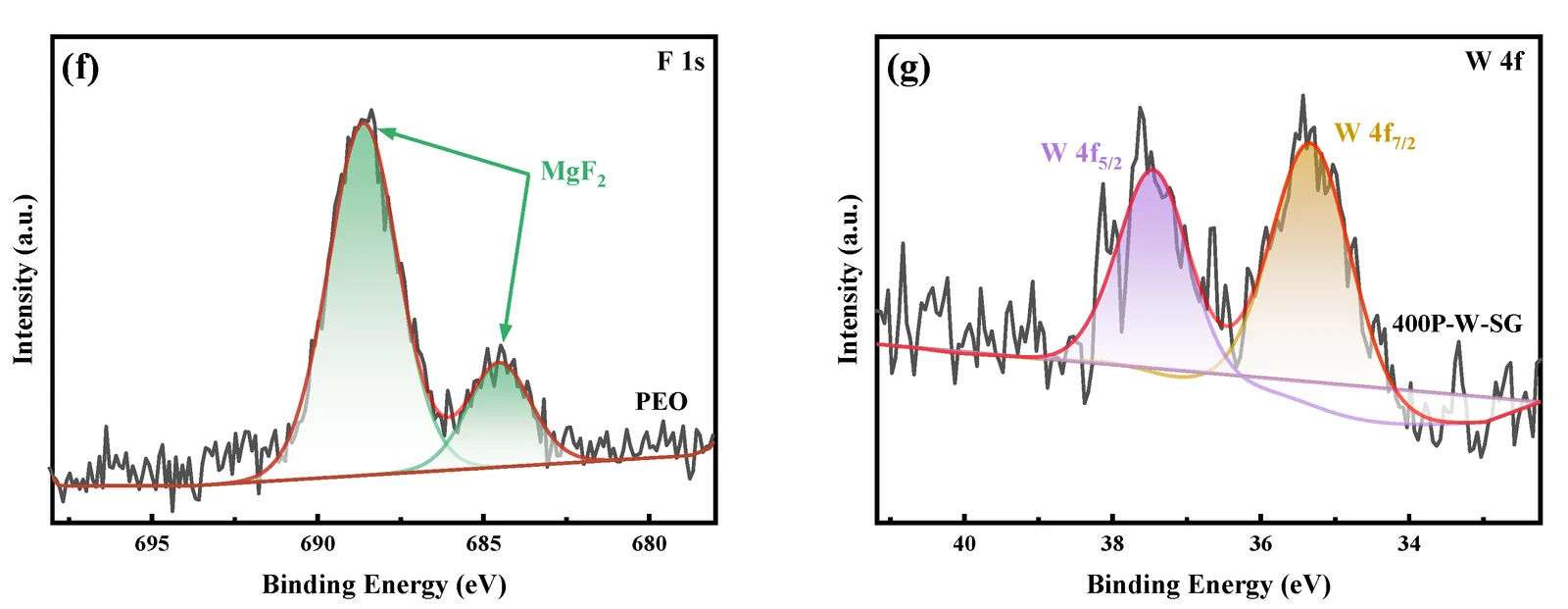
XPS spectra of 400-P coating and 400P-W-SG composite coating: (**a**) survey spectra, high-resolution spectra of (**b**) C1s, (**c**) Mg 1s, (**d**) O 1s, (**e**) Si 2p, (**f**) F 1s and (**g**) W 4f.

Figure 3 displays the survey spectra and high-resolution spectra of XPS for the 400-P coating and 400P-W-SG composite coating. As can be seen from Figure 3a, both the 400-P coating and the 400P-W-SG composite coating contain Mg, O, Si, F, and C elements. Among them, Mg comes from the AZ31B magnesium-alloy substrate, O, Si, and F come from the electrolyte. Additionally, the element W is detected in the 400P-W-SG composite coating. 

To further analyze the valence state and existence form of elements in the coatings, the high-resolution XPS spectra of the coatings are fitted and analyzed. In these specrea, the black lines represent the original data, while the red lines represent the fitted data. Figure 3b shows the detailed spectrum of C 1s. Compared with the 400-P coating, the C 1s in the 400P-W-SG composite coating is divided into three fitting peaks. The peaks at 284.8 eV and 288.4 eV represent foreign hydrocarbons, surface hydrocarbons, and CO_3_^2−^ [27], respectively, while the peak at 286.3 eV represents the C-O-C group, indicating that a layer of silane coating is successfully coated on the surface of the PEO coating, and the peak area at 286.3 eV is large, meaning that the silane coating is very thick.

In the Mg 1s spectrum, both the 400-P coating and the 400P-W-SG composite coating can fit a binding energy peak (Figure 3c), and after Na_2_WO_4_ impregnation and silanization treatment of the 400-P coating, the Mg 1s peak intensity of the obtained 400P-W-SG composite coating significantly decreases. This is attributed to the presence of a thick silane coating on the surface of the 400-P coating, which leads to weak signals detected for the 400-P coating during XPS testing. Similarly, as shown in Figure 3d, compared with the 400-P coating, the fine O 1s spectrum of the 400P-W-SG composite coating can fit two peaks. Among them, the peak at 531.9 eV is attributed to the main component MgO of the 400-P coating, while the larger area peak at 532.5 eV corresponds to C-O, which originates from the thick silane coating on the surface of the 400-P coating. In Figure 3e, in addition to the Mg_2_SiO_4_ peak at 102.1 eV in the 400-P coating, a SiO_2_ peak at 103.2 eV is also observed, and the peak corresponding to SiO_2_ in the 400P-W-SG composite coating is significantly higher than that in the 400-P coating. This observation suggests that a greater amount of SiO_2_ in the 400P-W-SG composite coating originates from the silane coating, implying that the silane coating on the 400-P coating is thicker. Figure 3f shows the F 1s spectrum, which originates from MgF_2_ in the 400-P coating, but MgF_2_ is not detected in the 400P-W-SG composite coating. This is attributed to its main presence in the dense layer inside the 400-P coating, and when the silane coating on the surface of the 400-P coating is thick, the F element in the 400P-W-SG composite coating cannot be detected. In Figure 3g, the characteristic peaks at 35.3 eV (W 4f_7/2_) and 37.5 eV (W 4f_5/2_) belong to the W^6+^ doublet [28], which prove that Na_2_WO_4_ is successfully loaded in the 400P-W-SG composite coating.

### 3.3. Surface Morphology and Elemental Composition and Distribution

**Figure 4:**
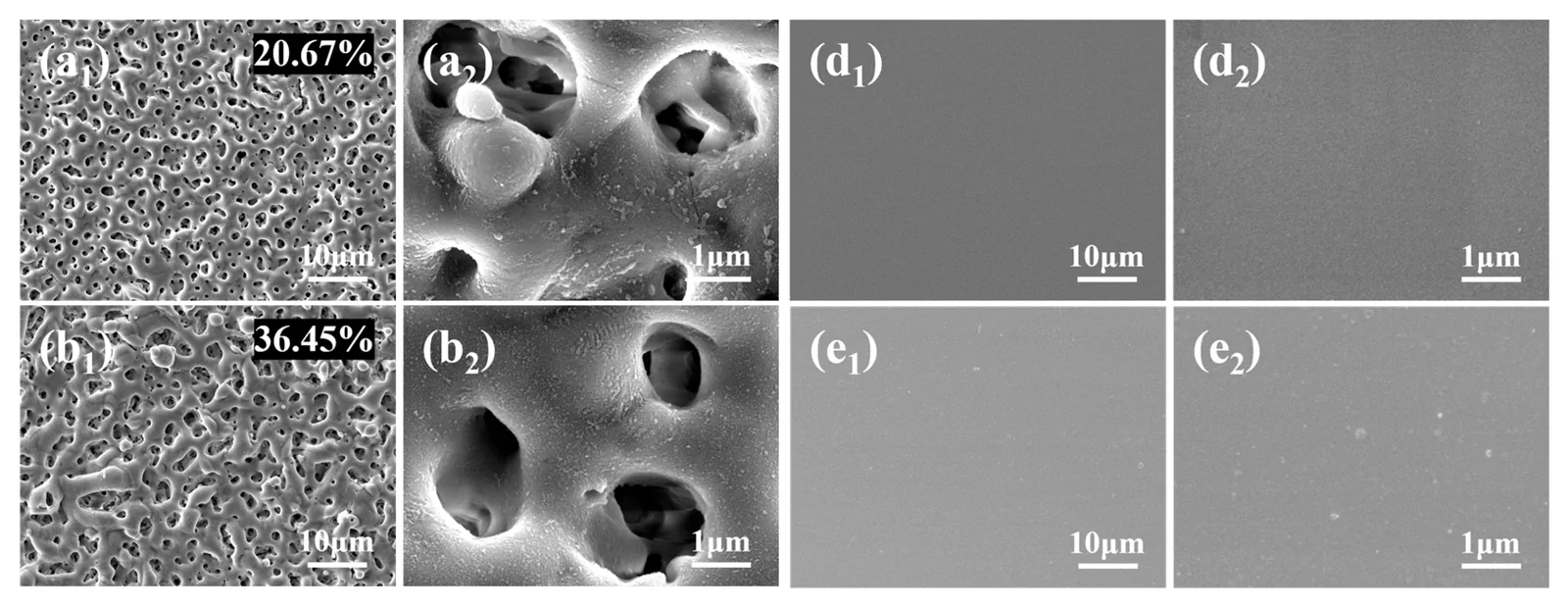

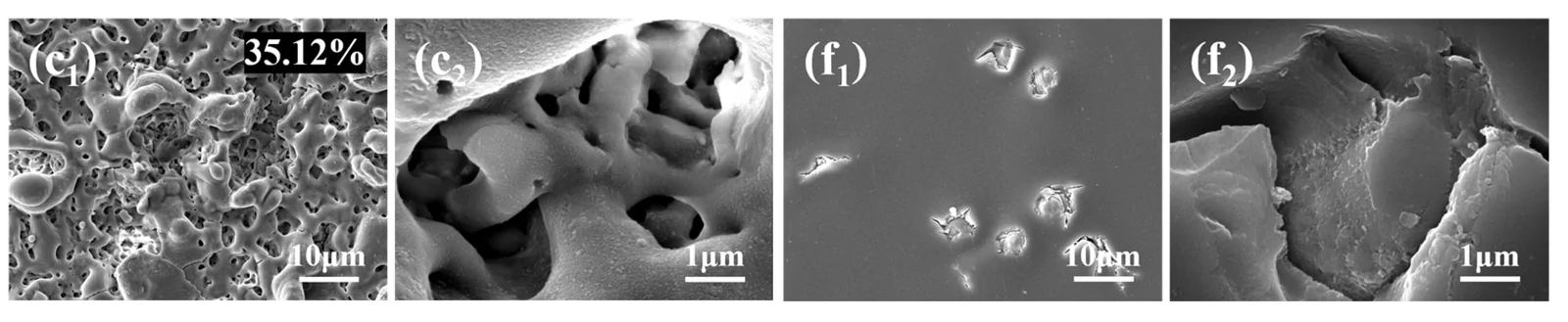
Surface morphologies of different coatings: (**a_1_**,**a_2_**) 300-P, (**b_1_**,**b_2_**) 350-P, (**c_1_**,**c_2_**) 400-P, (**d_1_**,**d_2_**) 300P-W-SG, (**e_1_**,**e_2_**) 350P-W-SG, (**f_1_**,**f_2_**) 400P-W-SG.

Figure 4 presents the surface morphology and surface porosity of different coatings. From Figure 4(a_1_–c_2_), it can be observed that with the increase in voltage, the surface morphology of PEO coatings exhibits different characteristics. The 300-P coating prepared at 300 V has more and smaller micropores, and their distribution is relatively uniform (Figure 4(a_1_)), and the coating presents a structure feature of pores within pores (Figure 4(a_2_)), which can provide effective space for the loading of Na_2_WO_4_. However, the porosity of the 300-P coating is relatively low, at 20.67%. Usually, more inhibitors can be loaded into larger and deeper pores [29]; therefore, a lower porosity means that less Na_2_WO_4_ can be loaded. From Figure 4(b_1_), it can be seen that the 350-P coating prepared at 350 V has a lower number of micropores compared to the 300-P coating, but the micropore diameters increase, resulting in a porosity of up to 36.45%, which can load more Na_2_WO_4_. When the voltage increases to 400 V, the prepared 400-P coating shows more obvious sintering and breakdown phenomena, and the surface becomes rough and uneven, with slight spalling at local areas, as shown in Figure 4(c_1_).

Compared with the 300-P and 350-P coatings, after loading Na_2_WO_4_ and silanization treatment, the surface of the obtained 300P-W-SG (Figure 4(d_1_,d_2_)) and 350P-W-SG (Figure 4(e_1_,e_2_)) composite coatings loses the typical porous structure of the PEO coating. The PEO coating is completely sealed by the silane coating, and the surface is dense and smooth. However, the 400P-W-SG composite coating prepared under a voltage of 400 V has defects such as pits on its surface (Figure 4(f_1_,f_2_)), indicating that loading Na_2_WO_4_ and silanization treatment have not effectively sealed the 400-P coating. Obviously, the microstructure of the PEO coating has a significant impact on the subsequent loading of Na_2_WO_4_ and silanization treatment effects.

Table 2 lists the elemental composition and content of different coatings. It can be seen from Table 2 that the PEO coatings prepared under different voltages are mainly composed of Mg, Al, F, O and Si elements. However, with the increase in voltage, the contents of O and Si elements in the coating increase, indicating that the coating become thicker. This is because higher voltages are more conducive to the formation of the PEO coating.

From Table 2 and Figure S1d–f, it can also be seen that for the PEO coatings with different structures prepared under different voltages, after loading Na_2_WO_4_ and silanization treatment, the obtained nP-W-SG composite coatings have extremely low amounts of Mg, Al, and F elements, and significantly increased contents of Si elements. Moreover, C and W elements are also present, and the mass percentage of C elements is higher than 38%. This indicates that a thick silane layer is formed on the surface of the PEO coating. With the increase in voltage, the content of W elements shows a trend of increasing first and then decreasing, which is consistent with the trend of surface porosity change, proving that a higher porosity is conducive to loading more Na_2_WO_4_. In addition, there is a local enrichment phenomenon of Mg elements in the 400P-W-SG composite coating (Figure S2f), which is in good agreement with the surface defects of the 400P-W-SG composite coating in Figure 4(f_1_), indicating that the microstructure of the PEO coating prepared under a higher voltage will affect the sealing effect of the silanization treatment.

### 3.4. Cross-Sectional Morphology and Element Composition and Distribution

**Figure 5:**
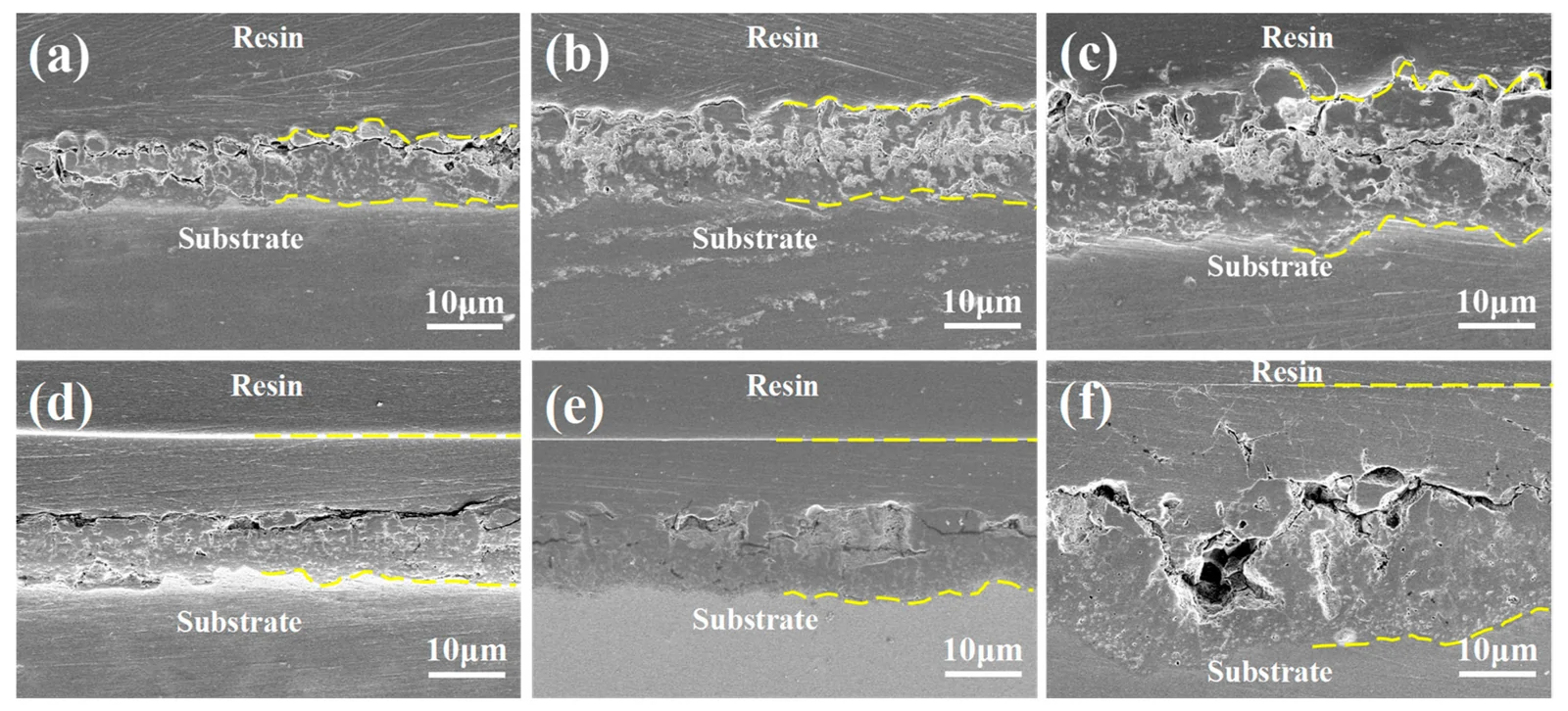
Cross-section morphology of different coatings: (**a**) 300-P, (**b**) 350-P, (**c**) 400-P, (**d**) 300P-W-SG, (**e**) 350P-W-SG, (**f**) 400P-W-SG.

**Figure 6:**
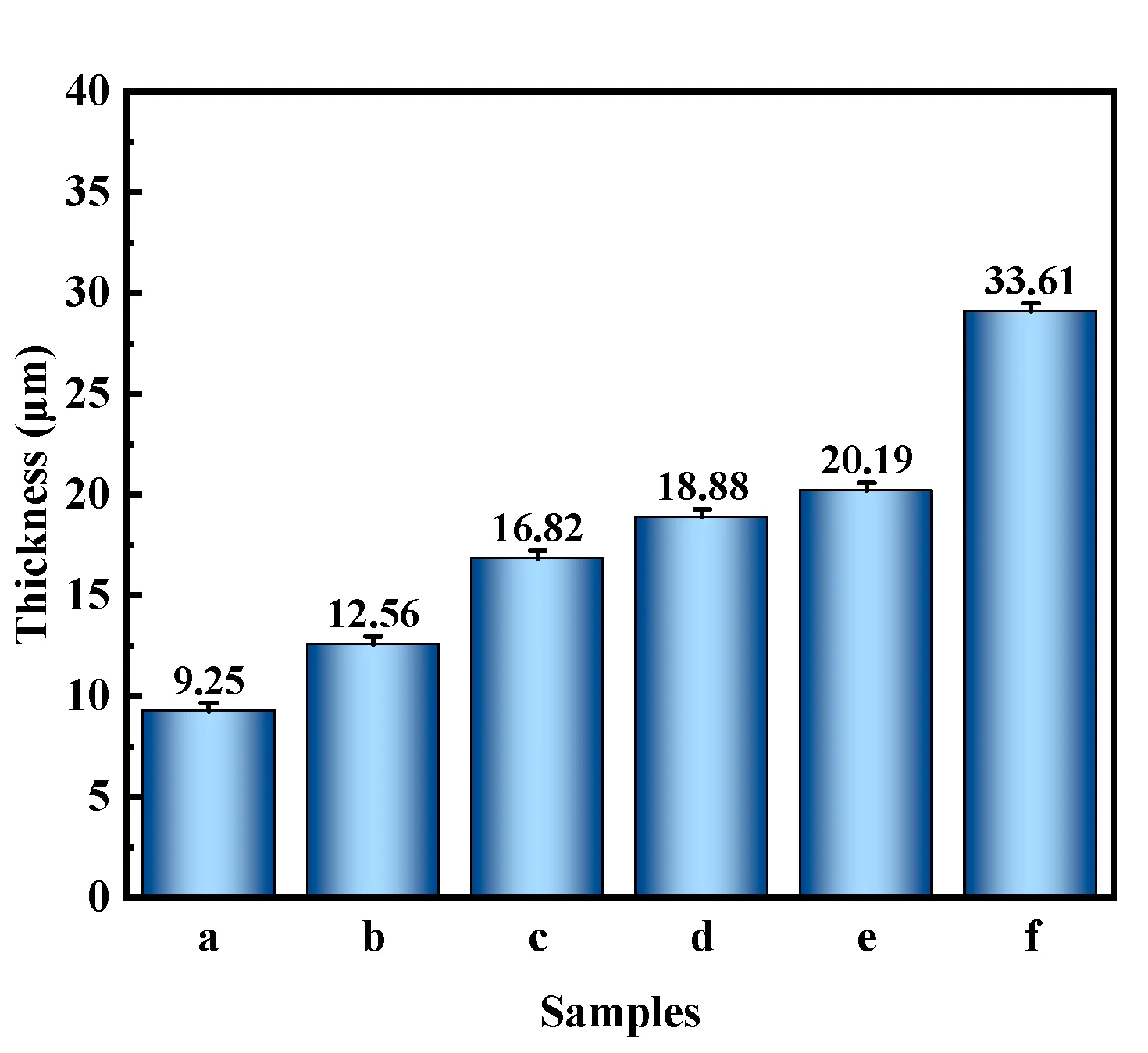
Thickness of different coatings: (**a**) 300-P, (**b**) 350-P, (**c**) 400-P, (**d**) 300P-W-SG, (**e**) 350P-W-SG, (**f**) 400P-W-SG.

Figure 5 and Table 3 show the cross-sectional morphology, as well as the composition and content of elements, of PEO and its composite coatings. The corresponding thickness is presented in Figure 6. From Figure 5a–c and Figure 6, it can be seen that as the voltage increases, the thickness of the PEO coating also gradually augments. When the voltage is 400 V, the prepared PEO coating has the largest thickness, approximately 16.82 μm. However, its external loose layer shows a poor uniformity and there are relatively obvious microcracks. In contrast, the 350-P coating shows the best uniformity and compactness. Furthermore, PEO coatings prepared at different voltages all contain Mg, Al, F, Si, and O elements (Table 3), and the F element is mainly distributed in the inner dense layer of the PEO coatings (Figure S3). This suggests that voltage has a negligible impact on the elemental composition and distribution of the coatings. However, as shown Table 3, with the increase in voltage, the contents of F, Si, and O elements rise, which is attributed to the increase in the thickness of the PEO coating. The C element comes from the resin material used when embedding the PEO coating.

After loading Na_2_WO_4_ onto the PEO coating and performing silanization treatment, an uniform, a dense and flat silane layer can be observed on the surface of the PEO coatings prepared under different voltages. Therefore, the thickness of the composite coatings is significantly thicker than that of the pure PEO coatings (Figure 5 and Figure 6). However, the 300P-W-SG composite coating (Figure 5d) exhibits poor bonding between the PEO coating and the silane layer. Similarly, the 400P-W-SG composite coating (Figure 5f) also shows analogous issues. A few voids are observed at the interface between the PEO coating and the silane layer. Specifically, the thickness non-uniformity of the 400-P coating leads to the silane coating’s inability to effectively remedy its micro-defects. In contrast, the 350P-W-SG composite coating shows a relatively dense cross-sectional morphology (Figure 5e), indicating that the microstructure of the PEO coating prepared at 350 V is conducive to improving the sealing effect of the silane layer on the PEO coating. In addition to the Mg, Al, F, Si, and O elements from the PEO coating, a clear and uniform Si element distribution layer is observed on the surface of the nP-W-SG composite coatings (Figure S3d–f). This again proves the existence of the silane layer on the surface of the PEO coating. Moreover, W can be detected in the nP-W-SG composite coatings, which indicates the successful loading of Na_2_WO_4_. The variation trend of the W element content is consistent with that of the porosity of the PEO coatings prepared under different voltages; that is, the 350P-W-SG composite coating exhibits the highest W element content, reaching 1.36 wt.% (Table 3). Therefore, when the PEO voltage is 350 V, the microstructure of the prepared PEO coatings has not only a favorable sealing effect of the silane coating on the PEO coating, but also a higher porosity that can load more Na_2_WO_4._

### 3.5. Corrosion Resistance

**Figure 7:**
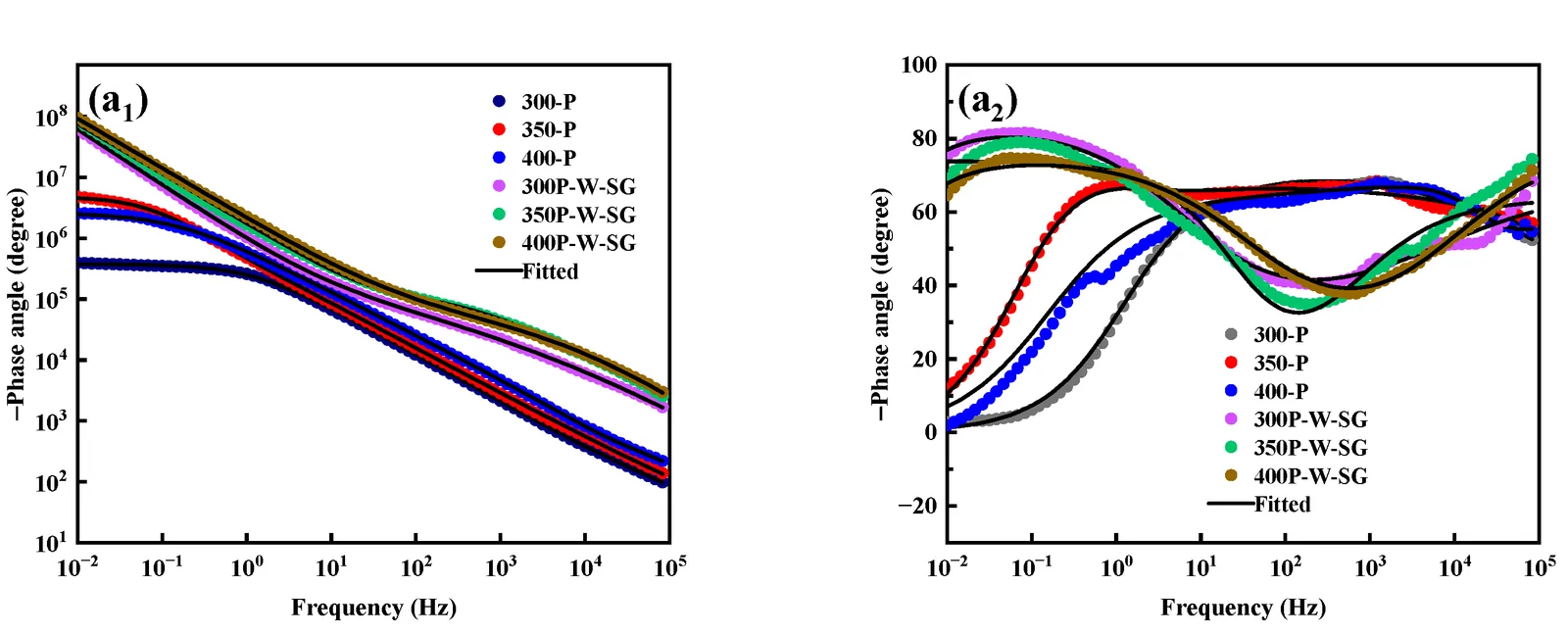

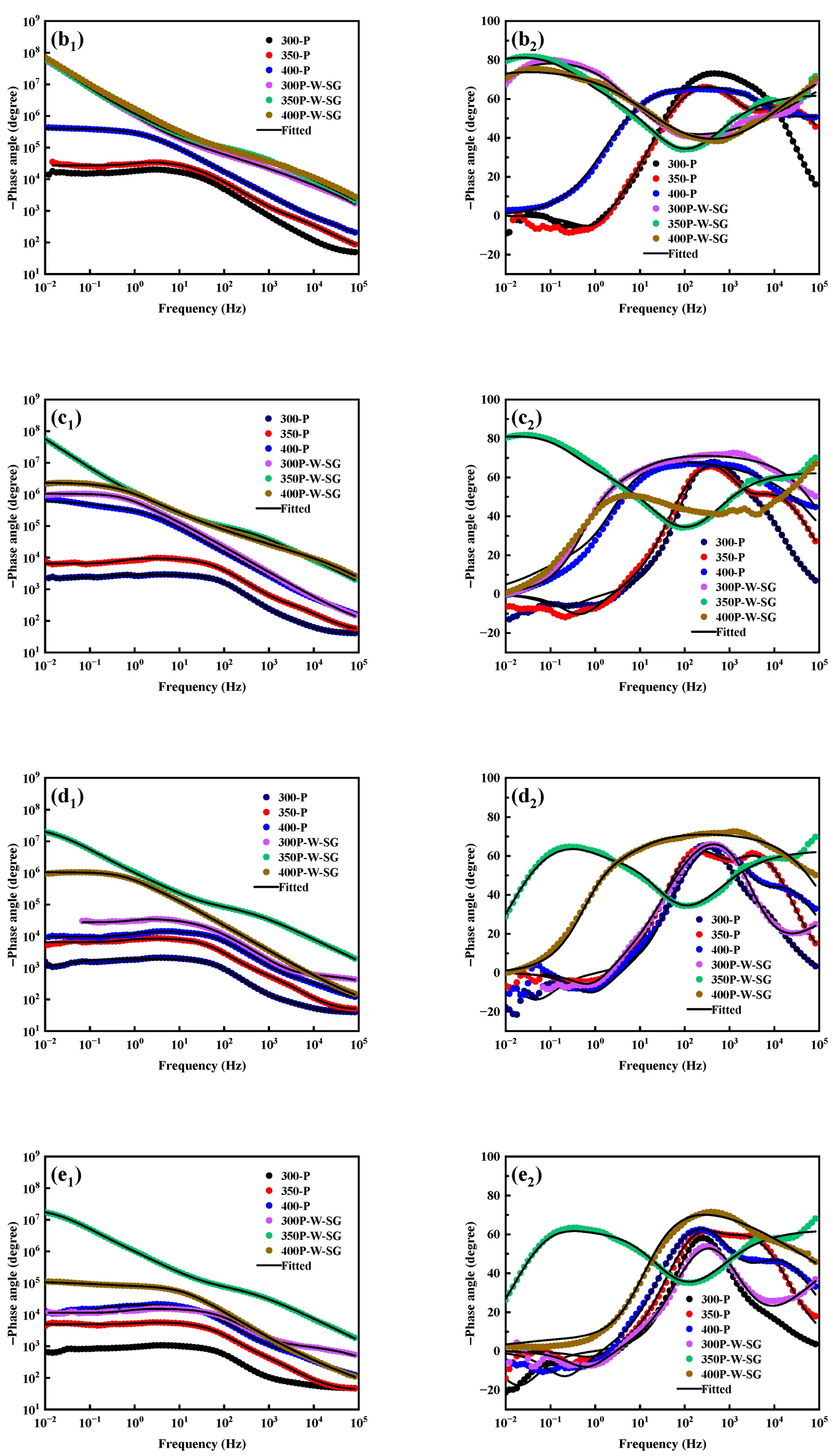

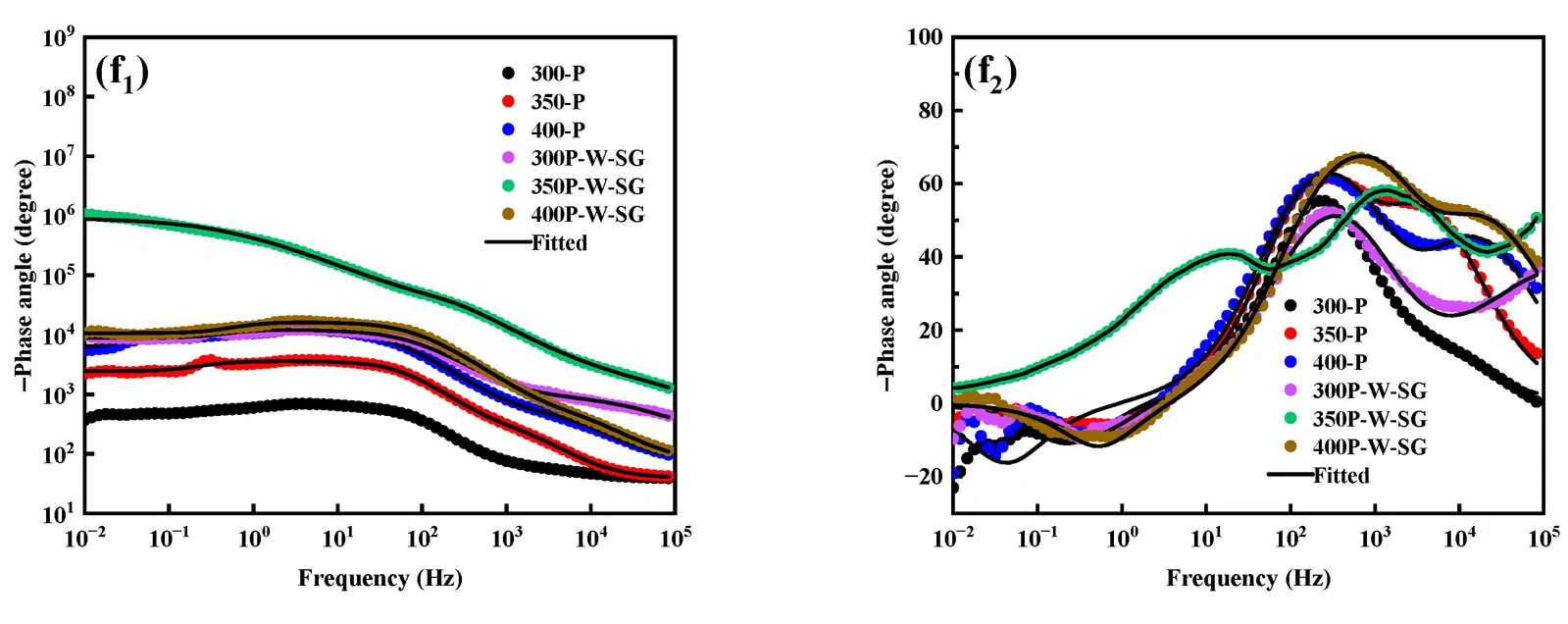
Experimental and fitted (**a_1_**–**f_1_**) modulus diagram and (**a_2_**–**f_2_**) phase-angle diagram of different coatings immersed in 3.5 wt.% NaCl corrosive media for different time: (**a_1_**,**a_2_**) 10 h, (**b_1_**,**b_2_**) 48 h, (**c_1_**,**c_2_**) 96 h, (**d_1_**,**d_2_**) 144 h, (**e_1_**,**e_2_**) 192 h, (**f_1_**,**f_2_**) 240 h.

**Figure 8:**
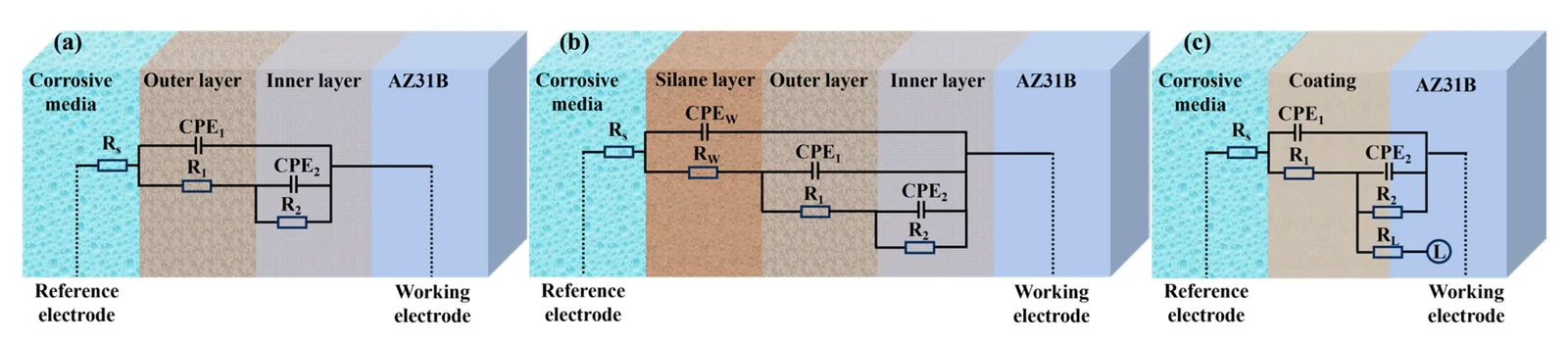
Equivalent circuit used to fit the electrochemical impedance spectra of PEO and its composite coatings prepared at different voltages: (**a**) fitting EIS having two time constants; (**b**) fitting EIS having three time constants; (**c**) fitting EIS having an induced reactance arc.

Figure 7 shows the electrochemical impedance spectra (EIS) and the corresponding fitting results of PEO and its composite coatings prepared under different voltages after immersion in 3.5 wt.% NaCl solution for 10 h, 48 h, 96 h, 144 h, 192 h and 240 h, respectively. Figure 8 displays the equivalent circuit used for fitting EIS, and the fitting results are listed in Table S1.

As can be seen from Figure 7(a_1_), after PEO coatings prepared at different voltages are immersed in the 3.5 wt.% NaCl solution for 10 h, and the impedance modulus of the 300-P coating is between 10^5^ and 10^6^ Ω cm^2^. With the increase in voltage, both the impedance modulus and the radius of the capacitive-reactance arc first increase and then decrease (Figure 7(a_1_) and Figure S4a). Specifically, the 350-P coating exhibits the largest impedance modulus and capacitive-reactance arc radius, thus showing the best corrosion resistance. This is attributed to the fact that the PEO coating prepared at 350 V has a greater thickness and a more uniform surface morphology. There are two time constants appearing in the corresponding phase-angle diagram (Figure 7(a_2_)). Therefore, the EIS of the PEO coating after 10 h of immersion can be fitted using the equivalent circuit shown in Figure 8a. In Figure 8a, R_s_ represents the solution resistance, and the resistance element R_1_ and its parallel constant phase-angle element CPE_1_ correspond to the outside loose layer of PEO coating, while the resistance element R_2_ and its parallel constant phase-angle element CPE_2_ correspond to the inside dense layer of PEO coating. No surface is an ideal interface due to heterogeneity and roughness, which is why the constant phase element (CPE) was chosen to interpret the EIS results instead of an ideal capacitor, determined by Equation (1) [19,23,24].

(1) $$Z_{CPE}=\left[\frac{1}{Y_{0}{\left(iw\right)}^{n}}\right]$$

where *Y*_0_ is the admittance of CPE and *n* is the frequency dispersion factor whose domain is 0–1, defining the ideality of the system in terms of being pure resistance (0) or pure capacitance (1). *ω* is the angular frequency (rad/s), and *i* = (−1)^0.5^.

**Figure 9:**
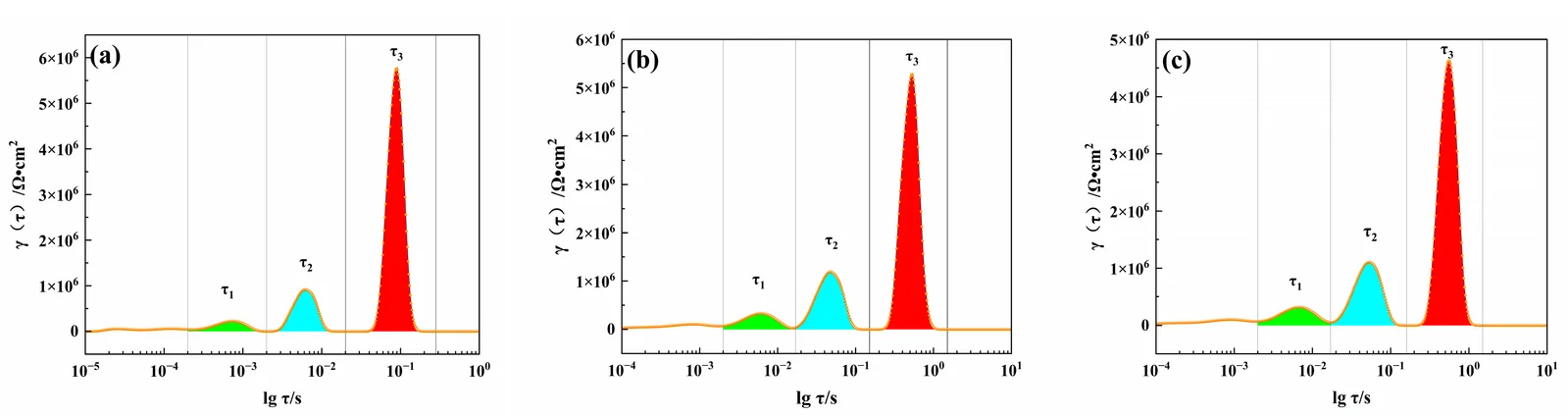
DRT plots of 350P-W-SG composite coating in long-term anticorrosion process: (**a**) 10 h, (**b**) 144 h, (**c**) 240 h.

After the PEO coatings prepared at different voltages are filled with Na_2_WO_4_ and subjected to silanization treatment, the impedance modulus of the resulting nP-W-SG composite coating reaches approximately 10^8^ cm^2^, which is 1 to 2 orders of magnitude higher than that of the simple PEO coating. The radius of the capacitive reactance arc also increases significantly (Figure S4a), indicating that loading Na_2_WO_4_ into the micropores of the PEO coating and silanizing the PEO coating are beneficial for enhancing the protective effect of the PEO coating on magnesium alloys. Moreover, the phase-angle curve of the nP-W-SG composite coatings exhibits three time constants (Figure 7(a_2_)); this can be confirmed by Figure 9a. In Figure 9, the horizontal axis represents the relaxation time *τ*, and the vertical axis represents the relaxation-time distribution function *γ*(*τ*). The relaxation time is the time required for a specific variable of the system to transition from a transient state to a steady state. In electrochemical systems, the relaxation time corresponds to the characteristic time constant, and its distribution corresponds to the distribution of characteristic time constants [30,31]. It can be seen from Figure 9a that when the 350P-W-SG composite coating is immersed for 10 h, its DRT curve can be divided into three peaks, marked as *τ*_1_, *τ*_2_, and *τ*_3_, respectively. Therefore, the equivalent circuit shown in Figure 8b is used to fit EIS of nP-W-SG composite coatings [32,33]. In Figure 8b, the resistance element *R_w_* and the constant phase-angle element *CPE_w_* represent the sodium tungstate layer sealed by a silane coating.

It can be seen from Figure 7(b_1_) that, after being immersed in a 3.5 wt.% NaCl solution for 48 h, the impedance moduli of the 300-P, 350-P, and 400-P coatings all decrease by one order of magnitude compared with those after 10 h of immersion. This indicates that the simple PEO coating is not conductively to the long-term corrosion resistance of the magnesium-alloy substrate. Low-frequency inductive arcs are observed in the Nyquist plots of the 300-P and 350-P coatings (Figure S4b), indicating that the corrosive medium has penetrated the coatings and corroded the substrate. Accordingly, the equivalent circuit depicted in Figure 8c is employed to fit the EIS at this stage. In Figure 8c, R_1_ denotes the coating resistance, which is connected in parallel with the constant phase element (CPE_1_). R_2_ represents the charge-transfer resistance, paralleled with the double-layer capacitor (CPE_2_). R_L_ refers to the resistance corresponding to pitting corrosion and is connected in series with the inductor (L). The 400-P coating still exerts a protective effect on the substrate after 48 h of immersion, which is attributed to the relatively large thickness of the PEO coating prepared at a voltage of 400 V. For the composite coatings fabricated via loading Na_2_WO_4_ and silanizing PEO coatings, the low-frequency impedance modulus of the obtained nP-W-SG composite coating remains almost unchanged after immersion in a 3.5 wt.% NaCl solution for 48 h. This indicates that within the 48 h immersion period, the corrosive medium has a relatively minor impact on the nP-W-SG composite coating.

It can be observed from Figure 7(c_1_) that, after 96 h of immersion, the impedance modulus values of the 400-P coating and the 350P-W-SG composite coating remain almost unchanged compared with those after 48 h of immersion, whereas the impedance modulus values of the 300-P and 350-P coatings decrease further. Meanwhile, the impedance modulus values of the 300P-W-SG and 400P-W-SG composite coatings decrease by approximately 1 and 2 orders of magnitude, respectively, compared with those after 48 h of immersion. This is because, for the 300P-W-SG composite coating, the PEO coating prepared at a voltage of 300 V is relatively thin; in contrast, for the 400P-W-SG composite coating, the silanization treatment does not completely seal the PEO coating prepared at a voltage of 400 V. Furthermore, the phase-angle curve of 300P-W-SG exhibits two time constants, indicating that the silane layer on the top of the 300P-W-SG composite coating may have dissolved or peeled off.

After 144 h of immersion, inductive reactance occurs in both the 400-P coating and the 300P-W-SG composite coating (Figure S4d). Additionally, the phase angle of the 400P-W-SG composite coating exhibits two time constants. After 192 h of immersion, the impedance modulus value of the 400P-W-SG composite coating remains at approximately 10^6^ cm^2^ (Figure 7(e_1_)). However, after 240 h of immersion, the 400P-W-SG composite coating exhibits inductive reactance (Figure S4f), with its impedance modulus value decreasing to about 10^4^ cm^2^ (Figure 7(f_1_)). In contrast, the impedance modulus value of the 350P-W-SG composite coating can still remain at 10^6^ cm^2^, and it is still controlled by a three-time constant response, as shown in Figure 7(f_2_) and Figure 9c, indicating that the silane layer on the surface of the 350P-W-SG composite coating still exists, thus demonstrating the best long-term corrosion resistance.

Furthermore, as can be seen from Table S1, under different immersion times, the R_2_ values of the PEO and its composite coatings are all higher than the R_1_ values. This indicates that throughout the immersion process, the internal dense layer of the PEO and its composite coatings plays a decisive role in protecting the substrate from the erosion of corrosive media. This can also be confirmed by Figure 9. In Figure 9, the relaxation time of the *τ*_1_ peak corresponds to the time constant of *R_W_*//*CPE_W_* in the equivalent circuit shown in Figure 8b; the relaxation time of the *τ_2_* peak corresponds to the time constant of *R*_1_//*CPE*_1_; and the relaxation time of the *τ_3_* peak corresponds to the time constant of R_2_//CPE_2_. Research shows that in the DRT diagram, the peak area within the relaxation time range (*τ*_1_, *τ*_1*+x*_) is positively correlated with the electrochemical impedance of the electrochemical dynamic process in the corresponding frequency range (*τ*_1*+x*_^−1^, *τ*_1_^−1^) [30,31]. In Figure 9, during the entire immersion process of the 350P-W-SG composite coating, the peak area of *τ_3_* is the largest (Table S2), indicating that the impedance of the inner layer of the coating is the greatest, thus showing that the inner layer of the coating always plays a crucial role in protecting the substrate from erosion by corrosive media.

**Figure 10:**
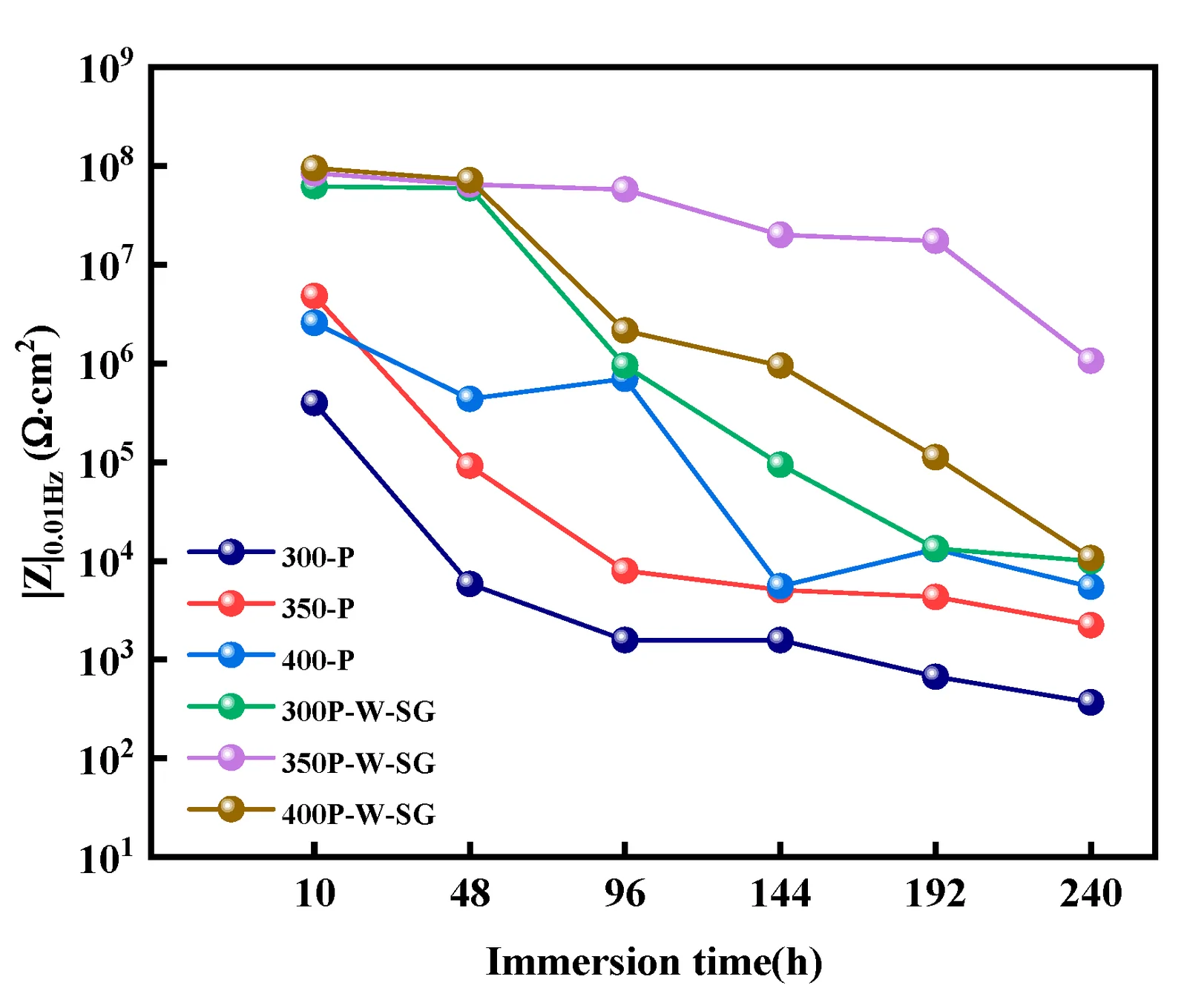
Low-frequency impedance modulus values of PEO and its composite coatings prepared at different voltages soaked in 3.5 wt.% NaCl solution for different time.

Figure 10 shows the low-frequency impedance modulus values (|Z|_0.01Hz_) of different coatings after being immersed in 3.5 wt.% NaCl solution for different durations. Research shows that the larger the |Z|_0.01Hz_, the better the overall corrosion resistance of the coatings [11]. Throughout the immersion process, the |Z|_0.01Hz_ of the 350P-W-SG composite coating is the largest, followed by 400P-W-SG, 300P-W-SG, 400-P, 350-P and 300-P in sequence. This indicates that the corrosion resistance of the simple PEO coating shows an increasing trend with the increase in voltage. Moreover, after Na_2_WO_4_ filling and silanization treatment, the corrosion resistances of the resulting composite coatings all increase. Among them, the corrosion resistance of the 350P-W-SG composite coating is the best.

However, as the soaking time increases, the |Z|_0.01Hz_ of the coating as a whole continuously decreases, indicating that the corrosion resistance of the coating gradually declines. The |Z|_0.01Hz_ of both 300-P and 350-P coatings shows a similar downward trend throughout the soaking period. When the 300-P coating is soaked for 48 h, its |Z|_0.01Hz_ drops to be close to that of the magnesium-alloy substrate [34], while the 350-P coating does not drop to a level close to that of the magnesium-alloy substrate until 96 h of soaking. This indicates that compared with the 300-P coating, the 350-P coating provides a longer protective effect for the magnesium-alloy substrate. During the soaking process, the |Z|_0.01Hz_ of the 400-P coating shows fluctuations, attributed to the formation and dissolution of corrosion products. At 144 h, the |Z|_0.01Hz_ of the 400-P coating drops to a level similar to that of the magnesium-alloy substrate, losing its protective effect on the substrate. When Na_2_WO_4_ is loaded on the surface of PEO coatings and then the coatings are treated with silane, the decrease magnitude of |Z|_0.01Hz_ of the prepared nP-W-SG composite coatings decreases significantly. Among them, the |Z|_0.01Hz_ of the 300P-W-SG composite coating drops to a level similar to that of the magnesium-alloy substrate after soaking for 192 h, and that of the 400P-W-SG composite coating after soaking for 240 h. However, after soaking for 240 h, the |Z|_0.01Hz_ of the 350P-W-SG composite coating is still as high as 1.06 × 10^6^ Ω·cm^2^, demonstrating the best long-term corrosion resistance.

### 3.6. Corrosion Morphology and Elemental Composition and Distribution

**Figure 11:**
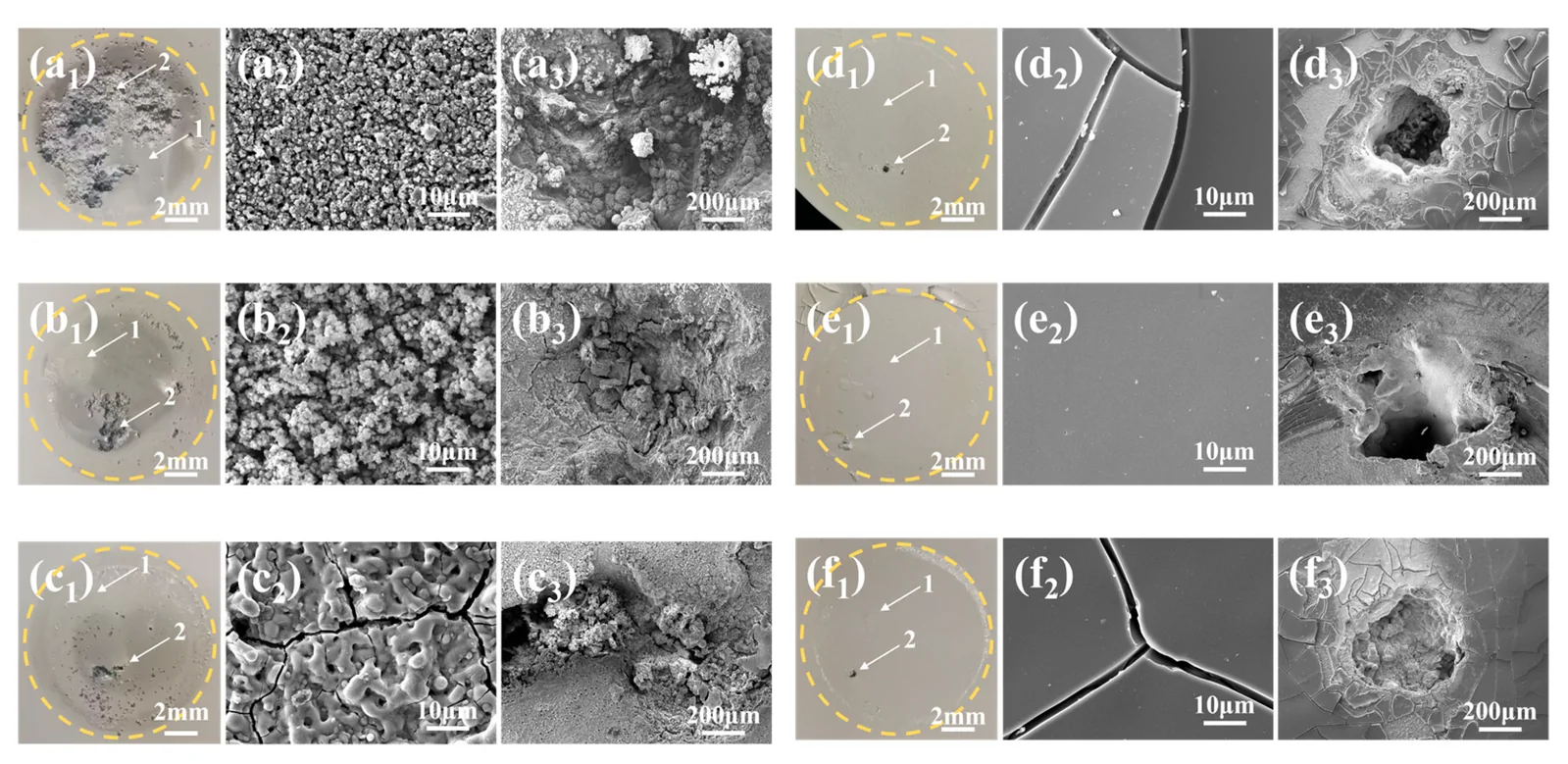
Macroscopic morphologies (**a_1_**–**f_1_**), microscopic morphologies of slightly corroded area (**a_2_**–**f_2_**) and microscopic morphologies of severely corroded area (**a_3_**–**f_3_**) of PEO and its composite coatings prepared at different voltages soaked in 3.5 wt.% NaCl solution for 240 h: (**a_1_**–**a_3_**) 300-P, (**b_1_**–**b_3_**) 350-P, (**c_1_**–**c_3_**) 400-P, (**d_1_**–**d_3_**) 300P-W-SG, (**e_1_**–**e_3_**) 350P-W-SG, (**f_1_**–**f_3_**) 400P-W-SG. Dashed circles in Figure 11a_1_–f_1_ show the exposed area (1 cm^2^), where 1 represents a slightly corroded area, and 2 indicates a severely corroded area.

Figure 11(a_1_–f_1_) presents the macroscopic morphology of PEO and its composite coatings prepared under different voltages after being immersed in 3.5 wt.% NaCl solution for 240 h. As can be seen from Figure 11(a_1_), the 300-P coating prepared under 300 V voltage is almost completely destroyed by the corrosive media during long-term immersion. With the increase in voltage, the area of corrosion pits on the surface of the PEO coatings gradually decreases. After filling the PEO coatings with Na_2_WO_4_ and silanization treatment, the area of the corrosion pit is further reduced. Among them, the 350P-W-SG composite coating (Figure 11(e_1_)) has the smallest corrosion area and the coating only shows slight bulge phenomena.

To further analyze the corrosion degree of the coating, the macroscopic slightly corroded areas (arrow 1 in Figure 11(a_1_–f_1_)) and severely corroded areas (arrow 2 in Figure 11(a_1_–f_1_)) are further analyzed microscopically. The results are shown in Figure 11(a_2_–f_2_,a_3_–f_3_).

From Figure 11(a_2_–c_2_), it can be seen that in long-term immersion, the macroscopic slightly corroded areas of the 300-P coating and 350-P coating are covered by loose corrosion-product layers, while the 400-P coating, due to its thicker thickness, only shows interlaced cracks in the slightly corroded area and still retains the typical basic structural characteristics of the PEO coating. When the PEO coatings prepared under different voltages undergo Na_2_WO_4_ loading and silanization treatment, cracks emerge on the surface of the 300P-W-SG composite coating (Figure 11(d_2_)) and 400P-W-SG composite coating (Figure 11(f_2_)). This phenomenon is likely attributed to the infiltration of corrosive media into the coating, which leads to uneven stress distribution both within and on the surface of the coating. However, the surface of the 350P-W-SG composite coating (Figure 11(e_2_)) is intact without corrosion or cracking of the silane coating.

From Figure 11(a_3_–c_3_), it can be observed that the severely corroded areas of PEO coatings prepared under different voltages all have obvious corrosion pits and a large amount of corrosion products, and the typical structure of the PEO coatings has disappeared. This indicates that the coating has peeled off and the corrosion media has penetrated into the magnesium-alloy substrate, suggesting that PEO treatment alone cannot achieve the long-term protective effect on magnesium alloys. After filling the PEO coatings with Na_2_WO_4_ and performing silanization treatment, the surface of the prepared nP-W-SG composite coatings shows that only obvious corrosion pits appear in the severely corroded areas (Figure 11(d_3_–f_3_)). The composite coating around the corrosion pits still exists, while varying degrees of corrosion products are accumulated within the pits. The presence of these corrosion products can to some extent slow down the corrosion of the magnesium-alloy substrate by the corrosion media.

In summary, with the increase in voltage, the corrosion degree of the coating decreases and the corrosion resistance of the coating increases. And after loading Na_2_WO_4_ on the PEO coating surface and performing silanization treatment, the corrosion degree of the coating further decreases and the corrosion resistance of the coating further increases.

## 4. Discussion

The plasma electrolytic oxidation process is a continuous process involving breakdown, reaction, condensation, and deposition. The voltage provides the driving force for the plasma electrolytic oxidation treatment process, which has a significant impact on the thickness and microstructure of the PEO coating. Generally, the higher the voltage, the stronger the electric-field strength and the greater the driving force of plasma electrolytic oxidation. This results in a higher reaction rate and faster coating growth. Therefore, the thickness of the PEO coating prepared at 400 V is 5.78 μm greater than that at 300 V and 4.27 μm greater than that at 350 V. However, the higher the voltage, the greater the breakdown discharge intensity, resulting in larger pores on the coating surface and rougher surface, as shown in Figure 4(a_1_–c_1_). The corrosion resistance of the PEO coating is mainly affected by many microscopic structural characteristic parameters such as the phase composition of the coating, thickness, surface defects, and compactness. With the increase in the treatment voltage, the phase composition of the coating remains unchanged. Although the number of micropores and the surface porosity of the coating increase, and the surface of the coating becomes rough and uneven, the greater thickness endows the coatings prepared at 350 V and 400 V with better corrosion resistance. As shown in Figure 7 and Figure 10, their low-frequency impedance modulus value |Z|_0.01Hz_ at the initial stage of immersion is approximately 1 order of magnitude higher than that at 300 V. In addition, compared with the PEO coating prepared at 300 V, the corrosion area of the PEO coatings prepared at 350 V and 400 V significantly decreases, and the corrosion degree lessens. Among them, the coating prepared at 400 V shows the smallest corrosion area and the slightest corrosion degree, as depicted in Figure 11(a_1_–c_2_).

When Na_2_WO_4_ is loaded onto the surface of PEO coatings prepared at different voltages and then undergoes silanization treatment, the content of Na_2_WO_4_ loaded on the coating surface varies. This variation is attributed to the distinct microstructures of the PEO coatings fabricated at different voltages. Among them, the surface porosity of the PEO coating prepared at 350 V is larger. The research shows that larger and deeper pores can hold more inhibitors [29]; thus, the content of Na_2_WO_4_ loaded into the micropores and onto the surface of the 350P-W-SG composite coating is the largest, as shown in Table 2 and Table 3. Na_2_WO_4_ is an environmentally friendly anodic corrosion inhibitor. When the corrosive media penetrates the coating and corrodes the substrate, it can combine with Mg^2+^ to form an insoluble Mg_2_WO_4_ protective layer (as shown in Equation (2)), thereby effectively preventing the further erosion of corrosive anions and H_2_O. The more Na_2_WO_4_ is loaded, the thicker the generated Mg_2_WO_4_ protective layer, the stronger the barrier effect against corrosive anions and H_2_O, and the better the corrosion resistance of the coating.

(2) $$Mg^{2+}+WO_{4}^{2-}\to Mg_{2}WO_{4}$$

**Figure 12:**
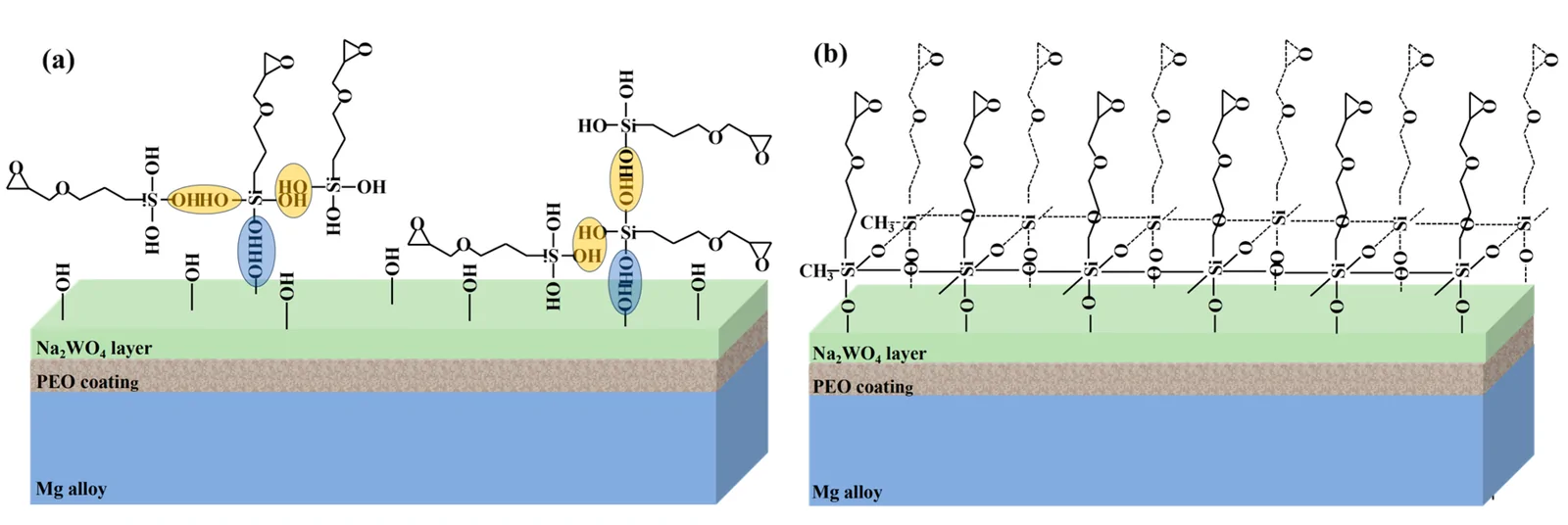
(**a**) Condensation reaction between pre-hydrolysis products of GPTMS molecules and hydroxyl groups on PEO coating surface, as well as self-condensation of GPTMS pre-hydrolysis products; (**b**) formation of silane layer.

The obtained nP-W-SG composite coatings are all composed of an internal PEO coating and an external Na_2_WO_4_ layer sealed by a silane coating. The silane coating is formed during the silanization process of the PEO coating surface. The pre-hydrolysis products of silane undergo condensation reactions with the hydroxyl groups on the surface of the PEO coating via Si-OH bonds, resulting in the formation of M-O-Si bonds (where M represents a metal atom). Simultaneously, condensation reactions also take place between the hydrolyzed silane molecules, forming Si-O-Si bonds, as illustrated in Equations (3) and (4) and Figure 12a,b. In this study, during the preparation of the silane solution, the aging time is set as long as 30 h. This extended aging period allows 3-(glycidyloxypropyl)trimethoxysilane (GPTMS) to fully hydrolyze and participate in condensation reactions. Consequently, the silane coating formed on the surface of the PEO coatings is relatively thick and compact, as depicted in Figure 4 and Figure 5.

(3) $${\left(CH_{3}O\right)}_{3}Si-{\left(CH_{2}\right)}_{3}-O-CH_{2}-C_{2}H_{3}O+3H_{2}O\to {\left(HO\right)}_{3}Si-{\left(CH_{2}\right)}_{3}-O-CH_{2}-C_{2}H_{3}O+3CH_{3}OH$$

(4) $$CH_{3}-Si{\left(OH\right)}_{3}+{\left(HO\right)}_{3}Si-O-CH_{2}-C_{2}H_{3}O\to \left(CH_{3}\right){\left(OH\right)}_{2}Si-O-Si{\left(OH\right)}_{2}-{\left(CH_{2}\right)}_{3}-O-CH_{2}-C_{2}H_{3}O+H_{2}O$$

**Figure 13:**
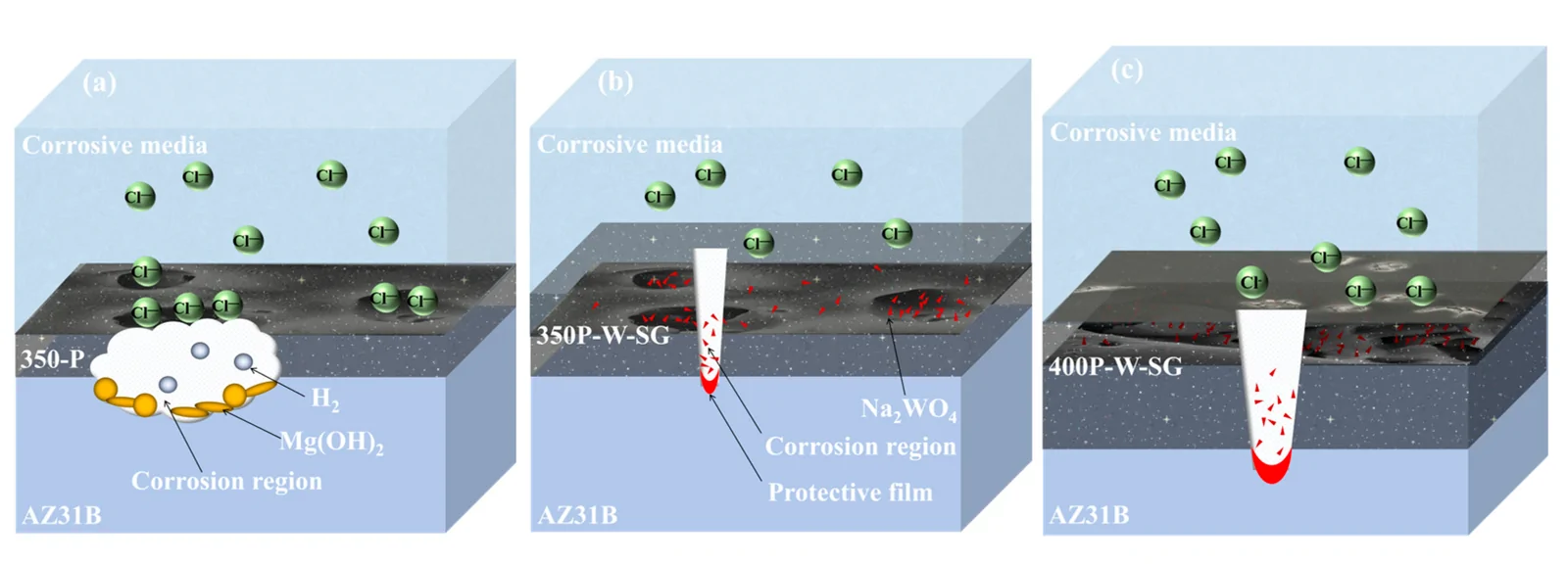
Schematic illustration of corrosion process of 350-P coating (**a**); 350P-W-SG composite coating (**b**); and 400P-W-SG composite coating (**c**) in 3.5 wt.% NaCl solution.

However, owing to the variations in voltage, the surface roughness and microstructure of the PEO coatings vary, resulting in silanization treatment having diverse sealing effects. Among them, the surfaces of the PEO coatings prepared at 300 V and 350 V are relatively smooth, so the resulting composite coatings are thick and dense. However, the PEO coating prepared at 400 V exhibits relatively high roughness, and even shows coating-peeling phenomena. This leads to incomplete sealing of the 400-P coating by the silane layer, as illustrated in Figure 4(f_1_,f_2_). Research indicates that in NaCl corrosive environments, the corrosive medium primarily corrodes the substrate by gradually penetrating the coating attributed to the high activity, small ionic radius, and strong penetration capability of Cl^−^ ions [35]. Consequently, a denser and thicker coating results in fewer and longer diffusion paths for the corrosive media to penetrate, prolonging the time required for the media to reach the substrate through the coating and thereby enhancing the coating’s corrosion resistance. Conversely, corrosive media can easily reach and corrode the substrate through defects in the coating. Therefore, the nP-W-SG composite coatings prepared under different voltages significantly enhance the corrosion resistance of PEO coatings, owing to their greater thickness and the slow-release effect of Na_2_WO_4_. The PEO composite coating prepared at 350 V (350P-W-SG) has the best corrosion resistance because the coating is thicker, the structure is denser, and the content of the loaded Na_2_WO_4_ is higher. In the early stage of immersion, the low-frequency impedance modulus |Z|_0.01Hz_ of the 350P-W-SG composite coating is about 1 order of magnitude higher than that of the PEO coating prepared at 350 V (350-P). After being immersed in 3.5 wt.% NaCl solution for 240 h, |Z|_0.01Hz_ of the 350P-W-SG composite coating is approximately 3 orders of magnitude higher than that of the 300P-W-SG composite coating. However, when the 400P-W-SG composite coating prepared at 400 V is immersed in a 3.5 wt.% NaCl solution, the incomplete sealing of the silane coating leads to the loss of loaded Na_2_WO_4_, thereby reducing its corrosion inhibition effect. Meanwhile, corrosive media can easily penetrate the coating through these defects to corrode the substrate. Therefore, although the pure 400-P coating exhibits the best overall corrosion resistance, after immersion in a 3.5 wt.% NaCl solution for 240 h, the |Z|_0.01Hz_ value of the 400P-W-SG composite coating is approximately 2 orders of magnitude lower than that of the 350P-W-SG composite coating. The schematic diagrams illustrating the possible corrosion processes of the single PEO coating (e.g., the 350-P coating), the 350P-W-SG composite coating, and the 400P-W-SG composite coating are shown in Figure 13a–c, respectively.

## 5. Conclusions

1. PEO coatings prepared at different voltages exhibit distinct microstructures: The 300 V coating (300-P) is thinner, with smaller micropores and lower porosity; the 350 V coating (350-P) is thicker, with uniformly distributed surface micropores and the highest porosity (36.45%); and the 400 V coating (400-P) has an uneven surface, inconsistent micropore size, non-uniform distribution, and high roughness, but its greater thickness endows it with the best corrosion resistance.
2. When Na_2_WO_4_ is loaded onto the micropores and surface of the PEO coating, followed by silanization treatment, the microstructure and corrosion resistance of the PEO coating are enhanced to varying extents. Specifically, the 350-P coating exhibits the highest porosity and the greatest Na_2_WO_4_ content. Consequently, the fabricated composite coating (350P-W-SG) features a dense and uniform structure. In contrast, the composite coating prepared at 400 V (400P-W-SG) has defects such as pits. This is attributed to the inherent surface imperfections of the 400-P coating. Therefore, the 350P-W-SG composite coating exhibits the best corrosion resistance due to its greater thickness, higher Na_2_WO_4_ content, and dense structure. After immersion in a 3.5 wt.% NaCl solution for 240 h, the 350P-W-SG composite coating still retains its integrity, and its low-frequency impedance modulus |Z|_0.01Hz_ reaches as high as 1.06 × 10^6^ Ω·cm^2^.
3. Further research is required to develop more corrosion-resistant and self-healing PEO-based composite coatings. This can be achieved by screening more effective single corrosion inhibitors or composite corrosion inhibitors, optimizing the preparation process parameters of the silane layer—such as selecting superior silane coupling agents, adjusting pH values, and controlling temperatures—or even replacing the sol–gel layer with highly corrosion-resistant and wear-resistant resin layers or layered double hydroxide layers.

## Figures and Tables

**Table 1:** Abbreviation of PEO coating and its composite coatings.

Ab.Name	Voltage/V	Loading of Na_2_WO_4_	Silanization Treatment
300-P	300	-	-
350-P	350	-	-
400-P	400	-	-
300P-W-SG	300	+	+
350P-W-SG	350	+	+
400P-W-SG	400	+	+

Note: “-” indicates not executed; “+” indicates executed.

**Table 2:** Surface elemental content of PEO coating and its composite coatings.

Sample	Mg (wt.%)	Al (wt.%)	F (wt.%)	O (wt.%)	Si (wt.%)	C (wt.%)	W (wt.%)
300-P	44.84	1.42	3.95	37.88	11.91	-	-
350-P	44.10	1.42	4.32	37.94	12.22	-	-
400-P	43.82	1.61	3.91	38.08	12.58	-	-
300P-W-SG	0.04	0.10	0.03	37.21	21.29	39.61	1.74
350P-W-SG	0.02	0.11	0.11	36.26	20.59	40.24	2.68
400P-W-SG	0.57	0.11	0.09	37.19	21.16	38.74	2.13

Note: “-” indicates no data.

**Table 3:** Elemental content of cross-section of PEO coating and its composite coatings.

Sample	Mg (wt.%)	Al (wt.%)	Si (wt.%)	O (wt.%)	F (wt.%)	C (wt.%)	W (wt.%)
300-P	36.49	0.69	0.82	10.40	0.90	50.69	-
350-P	33.58	0.49	1.23	14.63	1.85	48.22	-
400-P	30.67	0.70	2.01	18.73	2.20	45.69	-
300P-W-SG	34.32	0.62	2.96	15.71	1.62	43.95	0.82
350P-W-SG	41.59	0.72	4.77	12.85	0.87	37.85	1.36
400P-W-SG	27.27	0.48	6.81	23.80	2.72	37.60	1.32

Note: “-” indicates no data.

## Data Availability

The original contributions presented in this study are included in the article/Supplementary Material. Further inquiries can be directed to the corresponding authors.

## Supplementary Materials

The following supporting information can be downloaded at: https://www.mdpi.com/article/10.3390/ma18174146/s1, Figure S1: Composition of surface elements of different coatings: (a) 300-P, (b) 350-P, (c) 400-P, (d) 300P-W-SG, (e) 350P-W-SG, (f) 400P-W-SG; Figure S2. Distribution of surface elements of different coatings: (a) 300-P, (b) 350-P, (c) 400-P, (d) 300P-W-SG, (e) 350P-W-SG, (f) 400P-W-SG; Figure S3. Elemental distribution of cross section of different coatings: (a) 300-P, (b) 350-P, (c) 400-P, (d) 300P-W-SG, (e) 350P-W-SG, (f) 400P-W-SG; Figure S4. Experimental and fitted Nyquist diagram of different coatings immersed in 3.5 wt.% NaCl corrosive media for different time: (a) 10 h, (b) 48 h, (c) 96 h, (d) 144 h, (e) 192 h, (f) 240 h; Table S1. Fitted EIS data of PEO and its composite coatings in 3.5 wt.% NaCl corrosive media for different time; Table S2. DRT analysis of time constant area ratio during the long-term anti-corrosion process of 350M-W-SG composite coating.
