# Supplementary material for: Study on Loading of Na2WO4 and Silanization Treatment on Surface of Plasma Electrolytic Oxidation Coatings with Different Structures
Source: Materials (Basel). 2025 Sep 4;18(17):4146. doi: 10.3390/ma18174146 (PMC12430342; doi:10.3390/ma18174146)
Supplement: Supplementary file 1 [file materials-18-04146-s001.zip › materials-3812284-supplementary.pdf]

materials

MDPI

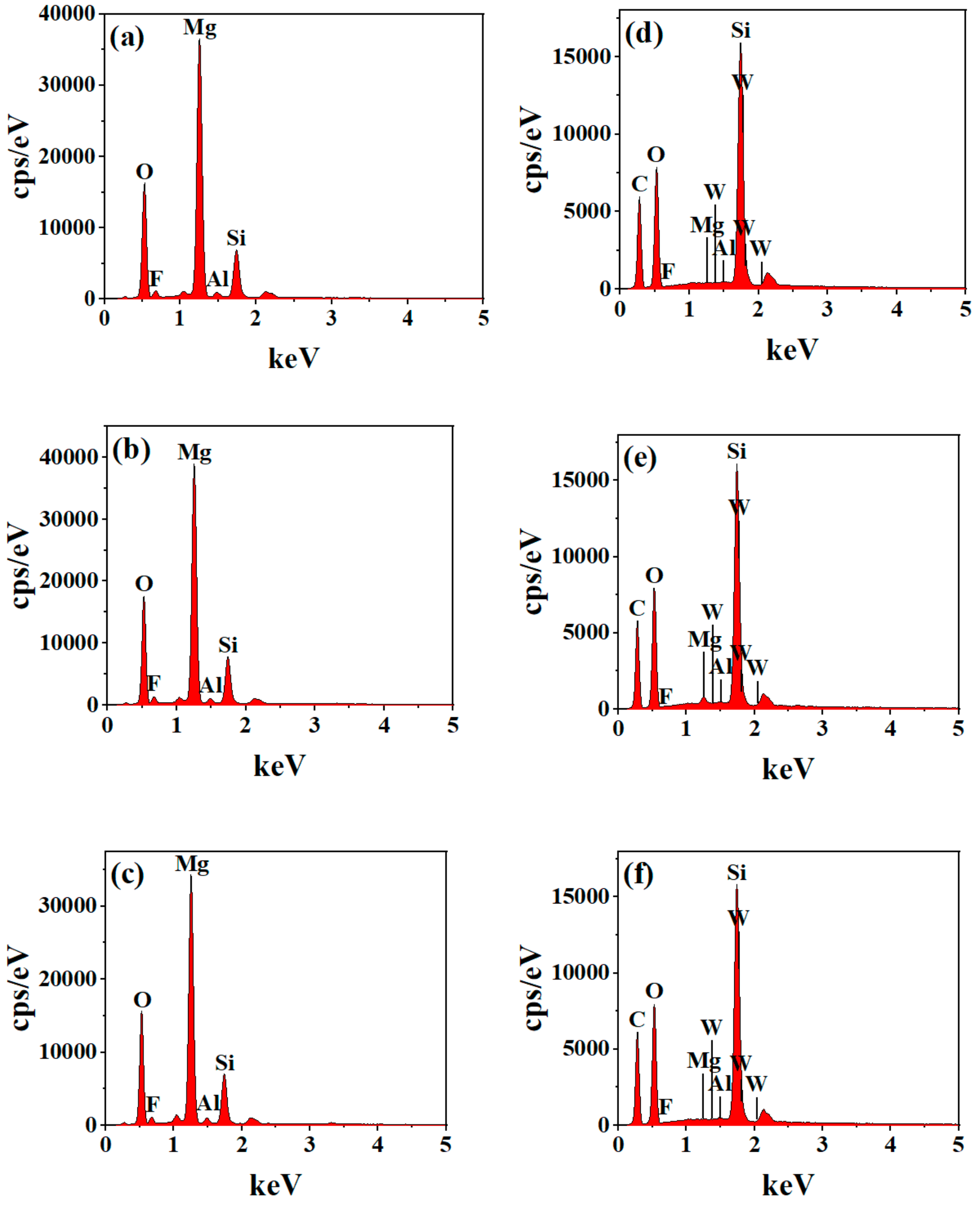

Figure S1. Composition of surface elements of different coatings: (**a**) 300-P, (**b**) 350-P, (**c**) 400-P, (**d**) 300P-W-SG, (**e**) 350P-W-SG, (**f**) 400P-W-SG.

 https://doi.org/10.3390/xxxxx

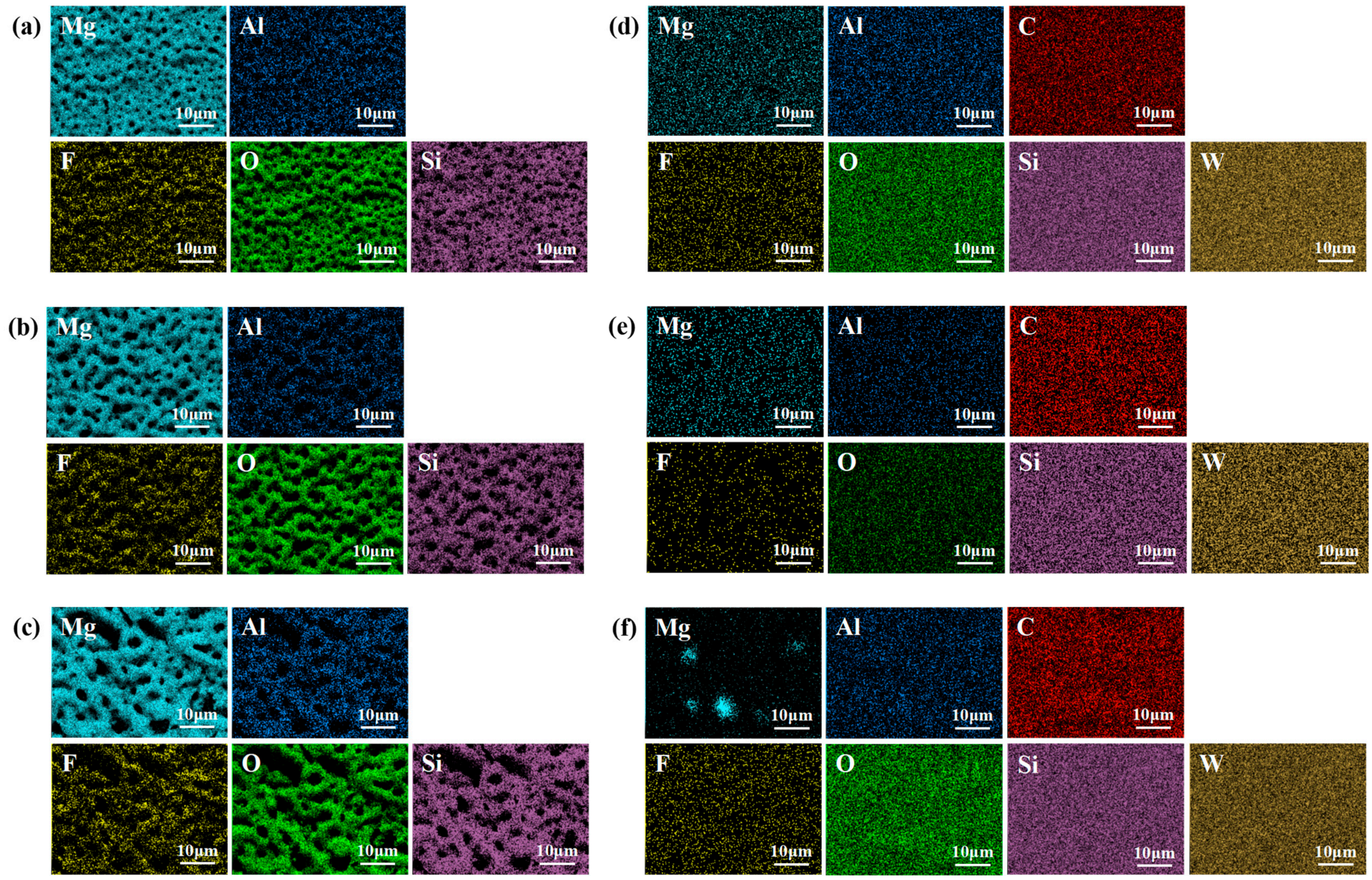

Figure S2. Distribution of surface elements of different coatings: (**a**) 300-P, (**b**) 350-P, (**c**) 400-P, (**d**) 300P-W-SG, (**e**) 350P-W-SG, (**f**) 400P-W-SG.

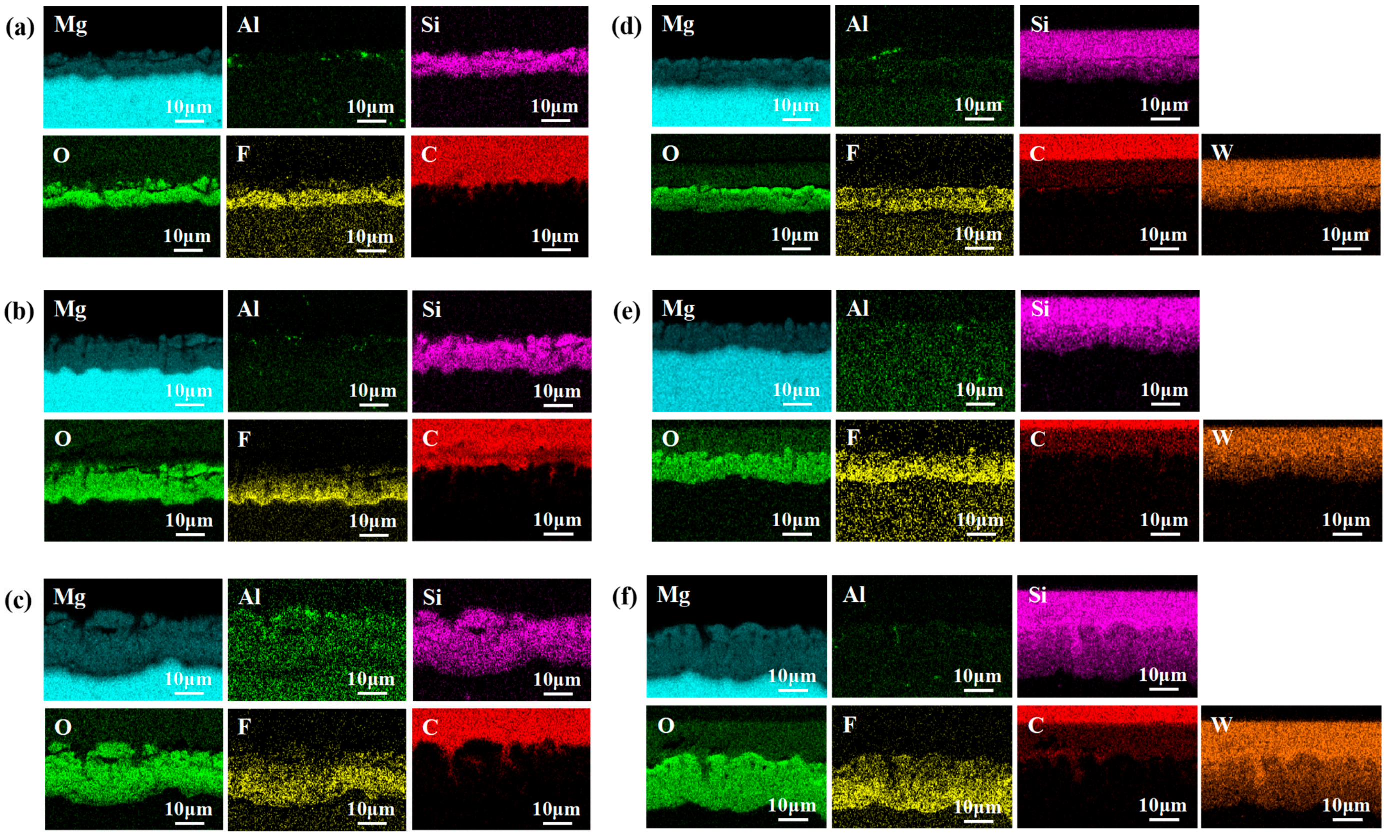

Figure S3. Elemental distribution of cross section of different coatings: (**a**) 300-P, (**b**) 350-P, (**c**) 400-P, (**d**) 300P-W-SG, (**e**) 350P-W-SG, (**f**) 400P-W-SG.

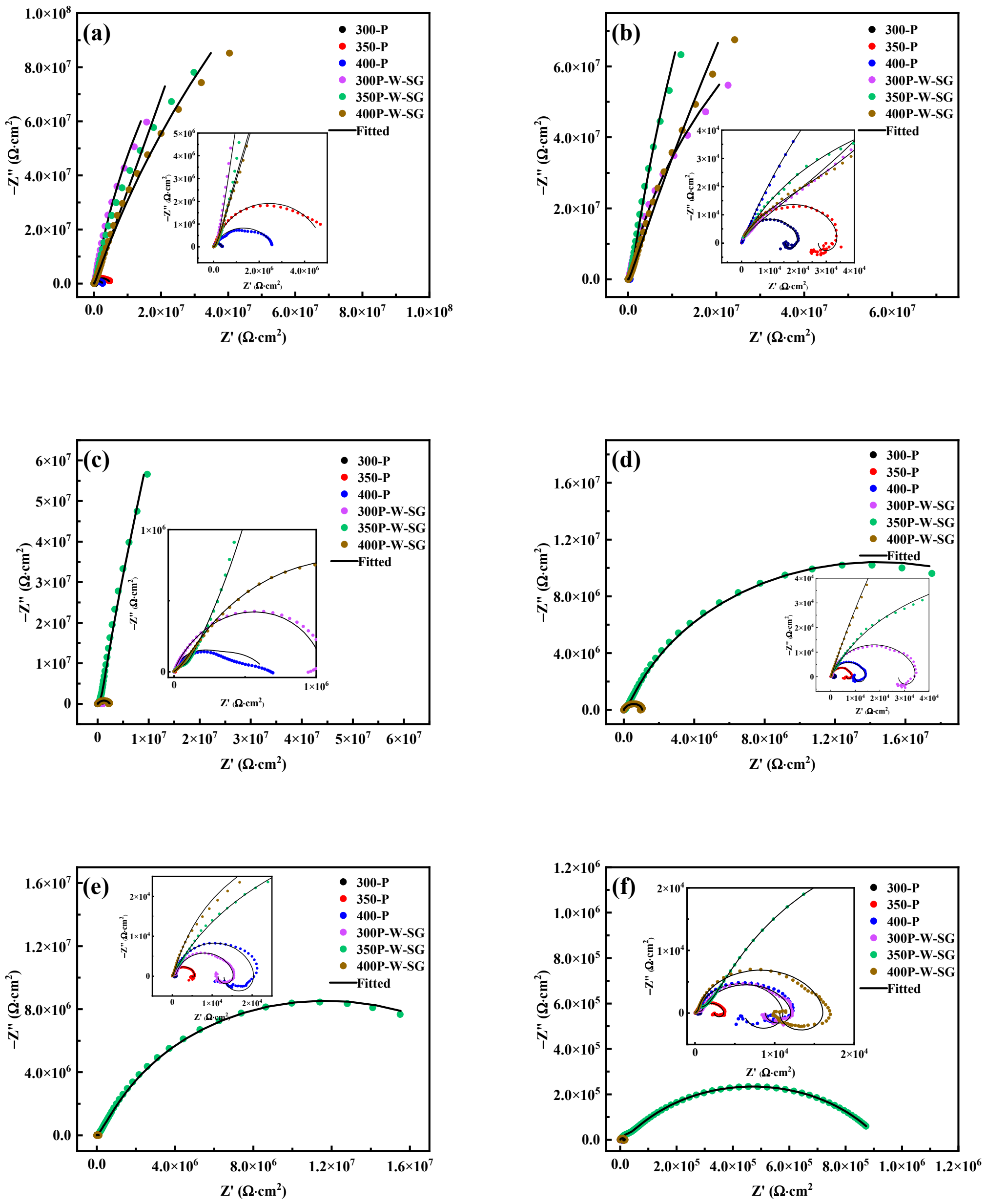

Figure S4. Experimental and fitted Nyquist diagram of different coatings immersed in 3.5 wt.% NaCl corrosive media for different time: (**a**) 10 h, (**b**) 48 h, (**c**) 96 h, (**d**) 144 h, (**e**) 192 h, (**f**) 240 h.

Table S1. Fitted EIS data of PEO and its composite coatings in 3.5 wt.% NaCl corrosive media for different time.

Sample	Immersion Time	R_w (Ω cm²)	CPE_w		R_1(Ω cm²)	CPE_1		R_2 (Ω cm²)	CPE_2		R_L	L	X^2 Value
			Y_0 (Ω^{-1} cm^{-2}· s^n)	n		Y_0 (Ω^{-1} cm^{-2}· s^n)	n		Y_0 (Ω^{-1} cm^{-2}· s^n)	n			
300-P	10 h	-	-	-	1.72×10^{5}	1.57×10^{-4}	0.40	3.67×10^{5}	5.22×10^{-7}	0.79	-	-	5.78×10^{-4}
350-P		-	-	-	8.18×10^{4}	1.85×10^{-6}	0.65	4.76×10^{6}	4.64×10^{-7}	0.86	-	-	9.74×10^{-4}
400-P		-	-	-	1.41×10^{3}	3.88×10^{-7}	0.71	2.65×10^{6}	4.65×10^{-9}	1	-	-	5.08×10^{-3}
300P-W-SG		8.53×10^{3}	2.55×10^{-8}	0.75	2.08×10^{6}	2.21×10^{-6}	0.73	7.85×10^{7}	2.09×10^{-7}	0.93	-	-	1.67×10^{-3}
350P-W-SG		9.66×10^{4}	3.80×10^{-8}	0.70	9.12×10^{6}	1.28×10^{-7}	0.70	1.06×10^{8}	2.96×10^{-8}	0.92	-	-	8.47×10^{-3}
400P-W-SG		8.73×10^{4}	2.95×10^{-6}	0.78	8.37×10^{6}	3.74×10^{-9}	0.86	9.77×10^{7}	1.05×10^{-7}	0.83	-	-	9.17×10^{-4}
300-P	48 h	-	-	-	3.43×10^{2}	1.61×10^{-7}	0.99	2.06×10^{4}	2.30×10^{-7}	0.80	5.80×10^{4}	1.37×10^{4}	1.22×10^{-3}
350-P		-	-	-	2.02×10^{3}	6.39×10^{-7}	0.76	3.55×10^{4}	5.32×10^{-8}	1	8.7×10^{4}	1.76×10^{4}	4.53×10^{-3}
400-P		-	-	-	1.83×10^{2}	2.78×10^{-7}	0.80	4.10×10^{5}	4.21×10^{-7}	0.76	-	-	6.98×10^{-4}
300P-W-SG		1.42×10^{3}	5.39×10^{-9}	0.86	2.00×10^{5}	1.02×10^{-6}	0.42	2.90×10^{6}	1.96×10^{-7}	0.90	-	-	1.68×10^{-3}
350P-W-SG		8.46×10^{4}	5.21×10^{-8}	0.71	6.93×10^{6}	5.20×10^{-7}	0.64	9.81×10^{7}	2.03×10^{-7}	0.93	-	-	1.67×10^{-3}
400P-W-SG		6.22×10^{4}	3.42×10^{-9}	0.87	6.41×10^{6}	2.02×10^{-6}	0.92	9.52×10^{7}	1.48×10^{-7}	0.84	-	-	8.70×10^{-4}
300-P	96 h	-	-	-	2.32×10^{2}	7.58×10^{-7}	0.94	2.61×10^{3}	3.65×10^{-7}	0.99	8.86×10^{3}	8.11×10^{3}	3.89×10^{-3}
350-P		-	-	-	7.47×10^{2}	6.90×10^{-7}	0.82	8.84×10^{3}	1.42×10^{-7}	0.82	1.62×10^{4}	6.86×10^{4}	3.77×10^{-3}
400-P		-	-	-	4.07×10^{5}	2.63×10^{-6}	0.63	2.41×10^{5}	4.14×10^{-7}	0.87	-	-	7.04×10^{-3}
300P-W-SG		-	-	-	1.73×10^{5}	5.78×10^{-7}	0.75	8.81×10^{5}	3.13×10^{-7}	0.92	-	-	1.54×10^{-3}
350P-W-SG		6.56×10^{4}	4.65×10^{-8}	1	4.76×10^{6}	1.88×10^{-7}	0.73	9.66×10^{7}	4.77×10^{-7}	0.62	-	-	1.48×10^{-3}
400P-W-SG		2.67×10^{2}	1.56×10^{-6}	0.68	1.65×10^{4}	4.87×10^{-7}	1	3.78×10^{6}	2.05×10^{-7}	0.78	-	-	8.49×10^{-4}
300-P	144 h	-	-	-	95.63	6.37×10^{-7}	0.99	1.76×10^{3}	1.23×10^{-6}	0.97	2.46×10^{3}	1.12×10^{4}	9.87×10^{-3}
350-P		-	-	-	1.04×10^{3}	4.66×10^{-7}	0.91	7.42×10^{3}	4.57×10^{-7}	0.93	1.92×10^{4}	6.62×10^{3}	3.02×10^{-3}
400-P		-	-	-	7.51×10^{2}	3.82×10^{-7}	0.81	1.37×10^{4}	1.12×10^{-7}	0.97	2.54×10^{4}	5.67×10^{3}	4.13×10^{-3}
300P-W-SG		-	-	-	7.18×10^{2}	9.05×10^{-7}	0.55	3.85×10^{4}	1.07×10^{-7}	0.96	9.19×10^{4}	1.97×10^{4}	2.37×10^{-3}
350P-W-SG		7.91×10^{4}	6.30×10^{-8}	0.69	1.95×10^{6}	1.82×10^{-7}	0.80	2.66×10^{7}	7.51×10^{-8}	0.81	-	-	1.32×10^{-3}
400P-W-SG		-	-	-	1.73×10^{5}	5.78×10^{-7}	0.75	8.81×10^{5}	3.13×10^{-7}	0.92	-	-	1.54×10^{-3}
300-P	192 h	-	-	-	50.94	9.37×10^{-7}	0.98	8.70×10^{2}	1.98×10^{-6}	0.98	8.14×10^{2}	9.85×10^{3}	5.76×10^{-3}
350-P		-	-	-	1.45×10^{3}	1.29×10^{-6}	0.84	4.32×10^{3}	2.39×10^{-7}	1	1.76×10^{4}	3.37×10^{3}	3.14×10^{-3}
400-P		-	-	-	1.12×10^{3}	4.04×10^{-7}	0.80	1.97×10^{4}	2.25×10^{-7}	0.93	2.92×10^{4}	2.79×10^{4}	4.47×10^{-3}
300P-W-SG		-	-	-	1.23×10^{3}	4.25×10^{-7}	0.61	1.47×10^{4}	2.26×10^{-7}	0.91	3.40×10^{4}	9.63×10^{3}	2.27×10^{-3}
350P-W-SG		7.79×10^{4}	6.66×10^{-8}	0.69	4.01×10^{5}	7.3410^{-7}	0.69	2.25×10^{7}	2.78×10^{-7}	0.82	-	-	1.06×10^{-3}
400P-W-SG		-	-	-	7.11×10^{4}	2.86×10^{-7}	0.90	4.45×10^{4}	2.74×10^{-6}	0.85	-	-	2.04×10^{-3}
300-P	240 h	-	-	-	33.56	1.76×10^{-6}	0.95	5.86×10^{2}	3.63×10^{-6}	0.96	1.05×10^{3}	7.62×10^{2}	7.59×10^{-3}
350-P		-	-	-	5.47×10^{2}	8.81×10^{-7}	0.90	3.03×10^{3}	4.54×10^{-7}	1	4.78×10^{3}	4.51×10^{3}	3.86×10^{-3}
400-P		-	--	-	6.25×10^{2}	2.50×10^{-7}	0.86	1.04×10^{4}	3.09×10^{-7}	0.95	1.18×10^{4}	6.17×10^{3}	9.15×10^{-3}
300P-W-SG		-	-	-	1.01×10^{3}	3.63×10^{-7}	0.66	1.13×10^{4}	2.87×10^{-7}	0.90	2.52×10^{4}	1.10×10^{4}	3.23×10^{-3}
350P-W-SG		1.35×10^{3}	8.03×10^{-7}	1	6.72×10^{4}	3.38×10^{-7}	0.84	9.03×10^{5}	5.41×10^{-7}	0.61	-	-	1.04×10^{-3}
400P-W-SG		-	-	-	1.07×10^{3}	2.20×10^{-7}	0.84	1.52×10^{4}	4.98×10^{-8}	1	9.35×10^{3}	9.36×10^{3}	1.82×10^{-3}

Note: "-" indicates no data.

Table S2. DRT analysis of time constant area ratio during the long-term anti-corrosion process of 350M-W-SG composite coating.

**Time/h**	τ_1	τ_2	τ_3
10	0.06%	0.14%	0.16%
144	1.55%	2.96%	3.21%
240	98.39%	96.89%	96.63%
